# Sugar based *N,N*′-didodecyl-*N,N*′digluconamideethylenediamine gemini surfactant as corrosion inhibitor for mild steel in 3.5% NaCl solution-effect of synergistic KI additive

**DOI:** 10.1038/s41598-018-21175-6

**Published:** 2018-02-27

**Authors:** Ruby Aslam, Mohammad Mobin, Jeenat Aslam, Hassane Lgaz

**Affiliations:** 1 Corrosion Research Laboratory, Department of Applied Chemistry, Faculty of Engineering and Technology, Aligarh Muslim University, Aligarh, 202002 India; 2 Laboratory of Applied Chemistry and Environment, ENSA, Ibn Zohr University, PO Box 1136, 80000 Agadir, Morocco; 3 Department of Applied Bioscience, College of Life & Environment Science, Konkuk University, 120 Neungdong-ro, Gwangjin-gu, Seoul 05029 South Korea

## Abstract

The inhibitory behaviour of non-ionic sugar based *N,N*′-didodecyl-*N,N*′-digluconamideethylenediamine gemini surfactant, designated as Glu(12)-2-Glu(12) on mild steel (MS) corrosion in 3.5% NaCl at 30–60 °C was explored using weight loss, PDP, EIS and SEM/EDAX/AFM techniques. The compound inhibited the corrosion of mild steel in 3.5% NaCl and the extent of inhibition was dependent on concentration and temperature. The inhibiting action of Glu(12)-2-Glu(12) is synergistically enhanced on addition of potassium iodide (KI) at all concentrations and temperatures. The inhibiting formulation comprising of 2.5 × 10^−3^ mM of Glu(12)-2-Glu(12) and 10 mM of KI exhibits an inhibition efficiency of 96.9% at 60 °C. Quantum chemical calculations and MD simulation were applied to analyze the experimental data and elucidate the adsorption behaviour and inhibition mechanism of inhibitors. MD simulation showed a nearly parallel or flat disposition for Glu(12)-2-Glu(12) molecules on the MS surface providing larger blocking area to prevent the metal surface from corrosion.

## Introduction

The significance of mild steel cannot be undervalued in the constructions and chemical industries based on their cost effectiveness, obtainability and immense mechanical properties. However, the poor corrosion resistance of mild steel limits its application^1–3^. One of the methods to efficiently control corrosion processes is the employment of corrosion inhibitors.

Several researchers have dealt with the synthesis and investigation of surfactants (cationic, anionic, non-ionic and zwitterionic) as corrosion inhibitors in various medium^4–9^. Non-ionic surfactants, which have no apparent charge on the head group, have notably lower critical micelle concentrations (CMCs) in comparison to the analogous ionic surfactants and are reported to possess high inhibition efficiencies for corrosion of metals in chloride media^10,11^. A novel class of surfactants called gemini, which consists of two hydrophobic chains and two polar/ionic head-groups united covalently by a rigid or flexible spacer has emerged during the last few years. When equated with single-head, single tail counterparts, these gemini surfactants offer better surface properties like much smaller CMC values, much greater efficiency in reducing surface tension than expected^12–14^ etc. Due to their greater performance, geminis have been generating increasing interest among researchers.

The surfactants as well as majority of the other inhibitors frequently employed in the industries are composed of some compounds that are unsafe and have been presently facing a lot of criticisms due to their threat to human and their environments. In the past two decade, the research in the field of ‘green’ corrosion inhibitors has been aimed toward the goal of using affordable, potent molecules with little or “zero” negative environmental impact^15,16^.

In view of the absence of toxicity, very good bio-degradability and dermatological compatibility, the use and production (industrial scale) of sugar-based surfactants has gradually increased in the recent past^17^. Furthermore, these surfactants also offer unexplored possibilities in the development of synthetic bio-molecules with unique and novel surface active properties.

One potential method to cost-effectively increase the corrosion inhibition efficiency is to employ the concept of synergism and utilize a combination of inhibitors^18,19^. The phenomenon of synergism in corrosion inhibition signifies the enhancement in the ability of an inhibitor to resist corrosion in the presence of secondary species in the corrosive medium. Halide ions are effective additives for surfactants in corrosion inhibition of steel in different media^20–24^.

In this paper, we are reporting the preparation of a sugar based non-ionic gemini surfactant, *N,N*′-didodecyl-*N,N*′-digluconamideethylenediamine, designated as Glu(12)-2-Glu(12). After confirming the structure of this sugar based non-ionic gemini surfactant, we evaluated its surface activities. A perusal of literature on corrosion inhibitors suggest that there is no published report on the corrosion inhibition effect of Glu(12)-2-Glu(12). Therefore, the present study was undertaken to assess the corrosion inhibition effect of, Glu(12)-2-Glu(12) gemini surfactant for mild steel in 3.5% NaCl solution using chemical and electrochemical techniques. Effect of electrolyte temperature (temperature range 30–60 °C) on corrosion inhibition of Glu(12)-2-Glu(12) has also been assessed and discussed. The associated activation energy of corrosion, enthalpy of activation, entropy of activation, and thermodynamic parameters such as equilibrium constant, standard free energy of adsorption, and entropy of adsorption were computed to elaborate the corrosion inhibition mechanism. Synergistic inhibition between Glu(12)-2-Glu(12) gemini surfactant and potassium iodide (KI) in 3.5% NaCl solution has also been investigated by means of weight loss and electrochemical methods, and the possible synergistic mechanism has been proposed. DFT calculations and MD simulation was employed in an attempt to understand the adsorption of Glu(12)-2-Glu(12) molecules on mild steel surface at the molecular level.

## Materials and Methods

### Materials and measurements

The tested material was commercially obtained mild steel, with the chemical composition (wt %), as analysed by optical emission spectrometer, was as follows: C-0.061, Mn-0.181, P-0.018, Cr-0.035, Mo-0.054, Al-0.017, V-0.034 and Fe-99.59. The mild steel was cut into 2.5 cm × 2 cm × 0.1 cm samples for weight loss measurements. For electrochemical measurements, circular specimens, with exposed surface area 1.0 cm^2^, were used. Then the samples were grated with 320#, 400#, 600# and 1200# abrasive papers in turn, rinsed with double distilled water and degreased with alcohol and acetone.

### Preparation of *N,N*′-didodecyl-*N,N*′-digluconamideethylenediamine, Glu(12)-2-Glu(12)

The gemini surfactant, Glu(12)-2-Glu(12), was synthesized in two steps following the procedure described in the literature^25^. Detailed experimental procedure described for the preparation of gemini surfactant and characterization are given as follows.

**Figure 1:**
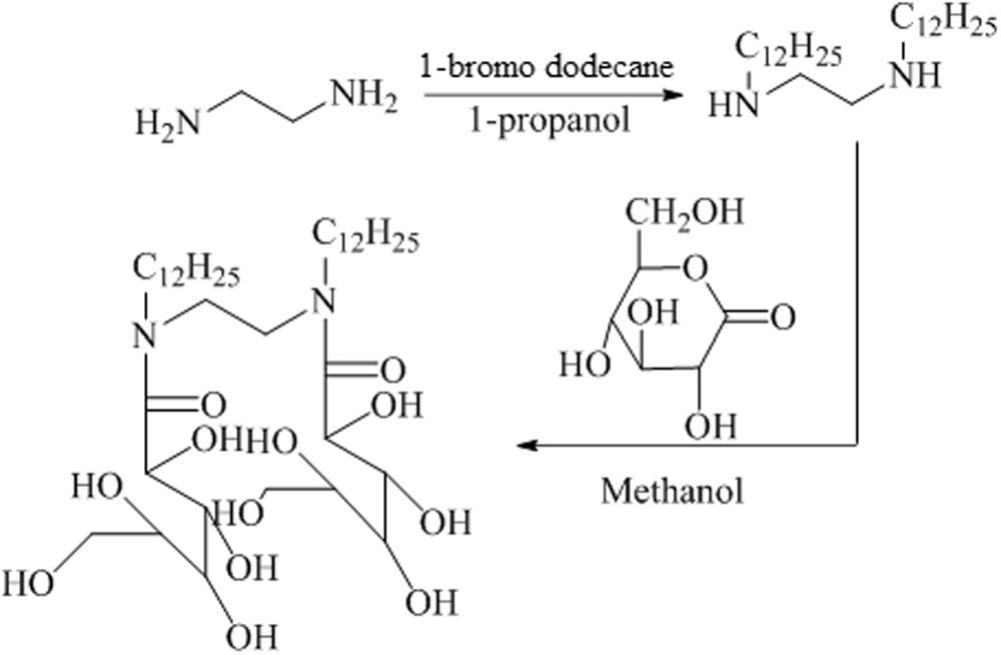
Pathway for the synthesis of Glu(12)-2-Glu(12) gemini surfactant.

Figure 1 shows the synthetic route of gemini surfactant. In the first step *N,N*′-dialkylethylenediamine was prepared by the reaction of ethylenediamine with 1-propanol and 1-bromododecane (added drop wise) under a mild alkaline condition with methanol NaOH solution. The mixture was refluxed for 24 h. After the filtrate was evaporated, the residue was poured in an aqueous NaOH solution and was stirred for 6 h. After filtration of the obtained crystalline white product from the diethyl ether solution, re-crystallization was performed three-times using a mixed solvent of ethanol containing a little amount of methanol. In the second step D-(+)-Glucono-1,5-lactone was added to a methanolic solution of *N,N*′-dialkylethylenediamine. The mixture was stirred for 1 week at room temperature, refluxed for 3 h and then evaporated. The obtained residue was washed with hexane three times to remove un-reacted *N,N*′-dialkylethylenediamine. After washing, the product was dissolved in dried acetone. Then the product was again washed with dried ethanol and finally, re-crystallized from a mixed solvent of dried acetonitrile containing a small amount of ethanol. The phlegmatic product was obtained.

The structure of the compound was confirmed by, ^1^H-NMR and FT-IR. The data is given below.

FT-IR (KBr, cm^−1^): 3421, 2924, 2836, 1623, 1498, 1073, 719.

^1^H-NMR (300 MHz, D_2_O): δ = 0.79–0.88 (t, 6 H,−2 × CH_3_, alkyl chain), 1.21–1.49 (m, 36 H, −2 × (CH_2_)_9_, alkyl chain), 1.61–1.71 (m, 4 H, −2 × NCH_2_CH_2_^−^), 3.275 (s, 12 H, −2 × N^+^(CH_3_)_2_), 3.81 (s, 4 H, −2 × CH_2_O), 4.31 (s, 4 H, −2 × N^+^CH_2_), 4.53 (s, 4 H, −2 × N^+^CH_2_COO).

### Surface tension measurement

Surface tension measurements were performed using a SD Hardson-Kolkata tensiometer by the ring detachment method. Prior to each experiment the instrument was calibrated at 30 °C with double distilled water. Requisite concentrations of Glu(12)-2-Glu(12) alone and in combination with 10 mM KI were obtained and after complete equilibration of solutions the readings were recorded. Force engaged in the complete detachment of Pt-Ir ring from the surface film was interpreted as surface tension, *γ* (mN/m). Measurement of *γ* was continued till the equilibrium values are attained. The particular concentration at which *γ* vs. concentration plot shows a break approaches to the CMC.

### Weight loss study

During weight loss study, the prepared mild steel specimens, were suspended in 3.5% NaCl solution in the absence and presence of different concentrations of Glu(12)-2-Glu(12) at 30, 40, 50 and 60 °C, respectively. The temperature was regulated by a thermostatic water bath. After 6 h immersion, the mild steel specimens were cleaned with a bristle brush, washed in double-distilled water and acetone, air-dried, and then reweighed.

The corrosion rate (ν, mg cm^−2^ h^−1^) and inhibition efficiency (*η*_w_%) at different concentrations were evaluated by equations (1) and (2), where *W*_0_ and *W* are the weight of samples before and after immersion in 3.5% NaCl solution, respectively, *A* is the total surface area of the samples, *t* is the immersion time, *ν*_0_ and *ν* are the corrosion rates in the absence and presence of the inhibitor, respectively. For weight loss measurements, triplicate experiments were performed simultaneously and the corrosion rates were averaged.

1 $$\nu =\frac{{W}_{0}-W}{At}$$

2 $${\eta }_{{\rm{w}}}( \% )=\frac{{\nu }_{0}-\nu }{{\nu }_{0}}\times 100$$

### Electrochemical measurements

The potentiodynamic polarization and electrochemical impedance (EIS) measurement tests were carried out using AUTOLAB instrument; model 128 N. A typical three electrode system from AUTOLAB was used for electrochemical tests. The system was composed of a silver/silver chloride as reference electrode, a platinum wire as counter electrode, and the mild steel sample with an exposed area of 1 cm^2^ as working electrode. Before the commencement of electrochemical tests, the working electrode was immersed in the electrolytic solution for 30 min for achieving a steady state potential. The electrochemical measurements were repeated thrice under identical experimental conditions.

During polarization experiments, the potential of the working electrode was varied from cathodic to anodic direction within a range of −250 to +250 mV with reference to open circuit potential (OCP) at a rate of 0.001 V/s. EIS measurements was carried out in the frequency range 10^5^ to 10^−2^ Hz at OCP. The sinusoidal potential perturbation was 10 mV in amplitude. The cell temperature was maintained at 30 ± 2 °C using a thermostatic water-bath.

The inhibition efficiencies (*η* %) were calculated by equations (3) and (4).

3 $${\eta }_{i}\,( \% )=\frac{{{i}}_{{corr}}-{{{i}}^{({\rm{i}})}}_{{corr}}}{{{i}}_{{corr}}}\times 100$$

where *i*_corr_ and *i*^(i)^_corr_ is corrosion current density without and with inhibitor, respectively and *η*_*i*_ (%) is the inhibition efficiency obtained by potentiodynamic polarization measurement.

4 $${\eta }_{R}\,( \% )=\frac{{{{R}}_{{ct}}}^{({\rm{i}})}-{{R}}_{{ct}}}{{{{R}}_{{ct}}}^{({\rm{i}})}}\times 100$$

where *R*_ct_ and *R*_ct_^(i)^ is the charge transfer resistance without and with inhibitor, respectively and *η*_R_ (%) is the inhibition efficiency obtained by EIS measurement.

### Surface characterization

In order to explore the relationship between the electrochemical behaviour and surface morphology, freshly polished mild steel coupons were suspended in 3.5% NaCl solution without and with optimum concentration of Glu(12)-2-Glu(12) for 6 h at room temperature and were examined by AFM and SEM/EDAX studies. The analysis were made using AFM-Dimension icon ScanAsyst equipped with Nanoscope V.

The SEM images were taken using a scanning electron microscope (Model: JEOL JSM- 6510LV) with EDAX attachment (Model: INCA, Oxford)

Details of operating mode of instruments and procedures for the surface analyses are reported somewhere else^20,26–28^.

### Quantum chemical calculations

DFT is a most widely accepted *ab initio* approach for modelling ground states of molecules and has been found to be successful in providing insights into the chemical reactivity and selectivity. DFT calculations were carried out using Gaussian 09 (Version D.01) program^29^ containing correlation functional of B3LYP (Lee-Yang-Paar) in addition to Becke three-parameter hydride functional using 6–31G (d, p), 6–311G (d, p) and 6–311G++ (d, p) basis sets^30,31^. All quantum calculations were carried out in aqueous phase using Self-Consistent Reaction Field (SCRF) theory, with Polarized Continuum Model (PCM)^32^. Electronic properties such as energy of the highest occupied molecular orbital (*E*_HOMO_), the energy of the lowest unoccupied molecular orbital (*E*_LUMO_), the energy gap, (Δ*E* = *E*_LUMO_ − *E*_HOMO_), ionization energy (*I*), electron affinity (*A*), electronegativity (*χ*), hardness (*η*) and fraction of electron transferred (Δ*N*) were calculated and recorded^33^.

## Molecular Dynamic (MD) Simulations

In order to model the adsorption behavior of tested inhibitor onto Fe (110) surface, MD simulations were carried out using discover module as implemented in Materials Studio 6.0 Software^34^. Fe (110) surface was selected for the simulation of the adsorption process. A supercell with a size of 25.06 × 25.06 × 25.14 Å^3^, contains 500 H_2_O and inhibitor molecule was created. This interaction simulation is performed in an area of (25.06 × 25.06 × 42.73Å^3^) with periodic boundary conditions and we studied five layers of iron to ensure that the surface depth was greater than the non-cut used in the calculation. The MD simulation is performed at 30 and 60 °C under canonical play (NVT) using a time step of 1.0 fs and a simulation time of 2000 ps. The details of the simulation process can be referred to our earlier papers^35,36^. All MD simulations were carried out with COMPASS force field^37^. Information about the interactions between the inhibitor with Fe (110) was determined by the calculation of the interaction and binding energies using equations^38^ (5) and (6)

5 $${E}_{{interaction}}={E}_{total}-({E}_{surface+solution}+{E}_{inhibitor})$$

6 $${E}_{binding}=-{E}_{{interaction}}$$

where *E*_total_ is the total energy of the entire system ${E}_{\mathrm{surface}+\mathrm{solution}}$ referred to the total energy of Fe (110) surface and solution without the inhibitor and ${E}_{{\rm{inhibitor}}}$ represent the total energy of inhibitor.

## Results and Discussion

### Surface active properties

Fig. S1 (supporting information) displays the surface tension (*γ*) of aqueous solutions of Glu(12)-2-Glu(12) as a function of concentration (log *C*). In the low surfactant concentration the *γ* decreases distinctly with increasing concentration and attains a break point. The intercept of two straight lines designates the critical micelle concentration (CMC).

The surface tension data shown in Fig. S1 (supporting information) allow us to calculate some physiochemical parameters such as effectiveness (*π*_CMC_), maximum surface excess (*Γ*_max_), minimum surface area (*A*_min_), standard free energy of micellization (Δ*G*^0^_mic_) and standard free energy of adsorption (Δ*G*^0^_ads_) at air/solution interface^39^ (see Table 1).

7 $${{\rm{\Pi }}}_{CMC}={\gamma }_{water}-{\gamma }_{CMC}.$$

8 $${{\rm{\Gamma }}}_{{\rm{\max }}}=-(\frac{1}{RT})\,(\frac{d\gamma }{d\,\mathrm{ln}\,C})$$

9 $${A}_{{\rm{\min }}}=(\frac{{10}^{20}}{{N}_{A}{{\rm{\Gamma }}}_{{\rm{\max }}}})$$

10 $${\rm{\Delta }}G{^\circ }_{{\rm{mic}}}=RT\,\mathrm{ln}\,{X}_{{\rm{CMC}}}$$

11 $${\rm{\Delta }}G{^\circ }_{{\rm{ads}}}={\rm{\Delta }}G{^\circ }_{{\rm{mic}}}-{{\Pi }}_{{\rm{CMC}}}/{{\Gamma}}_{max}$$

where *γ* = surface tension in mN m^−1^, *R* = gas constant (J mol^−1^ K^−1^), *T* = absolute temperature, (−*dγ/d* ln*C*) = slope of the plot of *γ* vs. log *C*, and *N*_A_ = Avogadro’ number.

**Table 1:** Surface active parameters of Glu(12)-2-Glu(12) and Glu(12)-2-Glu(12) + 10 mM KI in aqueous solution.

inhibitor	CMC × 10^−3^ (mM)	*γ*_CMC_ (mN/m)	*π*_CMC_ (mN/m)	*Г*_max_ (10^7^ × mol/m^2^)	*A*_min_ (Å^2^/molecule)	Δ*G*°_mic_ (KJ/mol)	Δ*G*°_ads_ (KJ/mol)
Glu(12)-2-Glu(12)	1.3	59.34	12.46	41.40	40.10	−16.75	−17.05
Glu(12)-2-Glu(12) + KI	0.61	45.43	26.37	41.81	39.70	−18.65	−19.28

CMC of the surfactants is looked as a key factor in the determination of their effectiveness as corrosion inhibitors^40^. For a surfactant to exhibit excellent corrosion inhibition, it should have a low CMC value as the effectiveness of inhibition of a surfactant decreases as the CMC value increases^30^. On the basis of this view, Glu(12)-2-Glu(12) + KI, which shows the low CMC value, may be considered as the effective corrosion inhibitor for mild steel in corrosive 3.5% NaCl solution. On comparing data obtained for Glu(12)-2-Glu(12) with that of Glu(12)-2-Glu(12) + KI, the presence of KI in Glu(12)-2-Glu(12) results in (i) a significant decrease in CMC, (ii) a decrease in *γ*_CMC_ (iii) higher value of *π*_CMC_ and (iv) the negatively higher value of ${\rm{\Delta }}{G}_{{\rm{ads}}}^{^\circ }$. The combination of these results advocates that the adsorption of the Glu(12)-2-Glu(12) + KI gemini surfactant at the air/aqueous solution interface occurs more effectively than for the Glu(12)-2-Glu(12). This necessarily leads to the formation of a tightly packed monolayer film of the longer chain analogue at the air/aqueous solution interface, which is supported by the greater *Γ*_cmc_ value and thereby, the smaller *A*_min_ value. We also note here that the calculated ${\rm{\Delta }}{G}_{{\rm{ads}}}^{^\circ }$ value of the Glu(12)-2-Glu(12) surfactant is negatively larger than the corresponding Δ*G*^°^_mic_ value, indicating that the adsorption occurs predominantly over the micellization that is seen in aqueous solution. This supremacy of adsorption has similarly been reported in the earlier literatures focusing on the adsorption/micellization behavior of sugar-based gemini surfactants^41^.

## Gravimetric Measurements

### Effect of concentration and temperature

The corrosion parameters of mild steel in 3.5% NaCl solution in the absence and presence of different concentrations of Glu(12)-2-Glu(12) and Glu(12)-2-Glu(12) + 10 mM KI are summarized in Table 2. It can be examined that *ν* of mild steel in presence of Glu(12)-2-Glu(12) or Glu(12)-2-Glu(12) +10 mM KI is both concentration and temperature dependent. With increase in Glu(12)-2-Glu(12) concentrations the corrosion rate decreases, which further decreases in presence of KI additive. This may be associated to the increased surface coverage of Glu(12)-2-Glu(12) at the mild steel/solution interface, which prevented the attack of 3.5% NaCl solution to mild steel surface and slowed down its dissolution. Glu(12)-2-Glu(12) in the presence of KI is observed to work more effectively. The improvement in the inhibition efficiency of Glu(12)-2-Glu(12) in the presence of KI can be explained on the basis of potential of zero charge and CMC lowering effect. The charge on the metal surface is determined from the value of E_corr_ − E_q=0_, where E_q=0_ is the zero charge potential. As reported in the literature, if E_corr_ − E_q=0_ > 0, the metal surface is positively charged^42^. In the present investigation, the obtained E_corr_ value of MS in 3.5% NaCl is −0.566 V (Ag/AgCl), whereas E_q=0_ of iron in NaCl solution is reported to be −0.659 V (Ag/AgCl)^43^. Thus the studied metal in NaCl solution is positively charged. The studied non-ionic surfactant is polar molecule having partial positive and partial negative sides. It has been proposed that halide ions are first adsorbed on the metal surface which leads to a recharging of the electrical double layer and the inhibitor is dragged into the double layer by electrostatic interaction with the adsorbed halide ions, forming ion-pairs directly on the surface of the metal. This ion-pair interaction consequently increases the surface coverage thereby reducing metal dissolution. The improvement in the inhibition efficiency of the studied inhibitor in presence of KI may be further explained on the basis of CMC lowering effect of gemini surfactant by the added salt^44^. Generally, the surfactant having lower CMC values has greater adsorption capability^45,46^. The addition of salt to the surfactants is known to decrease their solubility in the solution resulting in the formation of micelles at lower concentration and thus affecting the inhibition performance.

**Table 2:** Weight loss measurement results for MS in 3.5% NaCl solution in the absence and presence of different concentrations of Glu(12)-2-Glu(12) and Glu(12)-2-Glu(12) + 10 mM KI at temperatures 30–60 °C.

Glu(12)-2-Glu(12) (mM)	KI(mM)	*ν* (mg cm^−2^ h^−1^)				*η*_w_ (%)				*S*_θ_			
		30 °C	40 °C	50 °C	60 °C	30 °C	40 °C	50 °C	60 °C	30 °C	40 °C	50 °C	60 °C
3.5% NaCl		0.056 ± 0.001	0.224 ± 0.011	0.38 ± 0.022	1.504 ± 0.088	—	—	—	—	—	—	—	—
1 × 10^−5^	—	0.041 ± 0.002	0.149 ± 0.008	0.236 ± 0.006	0.878 ± 0.044	27.1	33.5	37.9	41.6	—	—	—	—
5 × 10^−5^	—	0.035 ± 0.001	0.134 ± 0.005	0.203 ± 0.009	0.698 ± 0.029	37.4	40.2	46.6	53.6	—	—	—	—
1 × 10^−4^	—	0.029 ± 0.001	0.107 ± 0.005	0.168 ± 0.008	0.563 ± 0.033	47.9	52.7	55.7	62.7	—	—	—	—
5 × 10^−4^	—	0.024 ± 0.001	0.086 ± 0.003	0.132 ± 0.005	0.447 ± 0.022	56.5	61.7	65.4	70.3	—	—	—	—
1 × 10^−3^	—	0.021 ± 0.001	0.065 ± 0.003	0.096 ± 0.004	0.266 ± 0.010	63.4	71.1	74.7	82.3	—	—	—	—
1.5 × 10^−3^	—	0.018 ± 0.001	0.053 ± 0.001	0.079 ± 0.005	0.178 ± 0.007	67.8	76.1	79.2	88.1	—	—	—	—
2.5 × 10^−3^	—	0.015 ± 0.001	0.047 ± 0.002	0.069 ± 0.003	0.12 ± 0.006	69.1	78.9	81.7	92.0	—	—	—	—
0	10	0.050 ± 0.001	0.045 ± 0.001	0.042 ± 0.002	0.04 ± 0.002	10.9	18.0	24.1	29.7	—	—	—	—
1 × 10^−5^	10	0.032 ± 0.001	0.117 ± 0.006	0.49 ± 0.029	0.64 ± 0.032	42.6	46.4	49.2	56.8	1.1	1.1	1.0	1.1
5 × 10^−4^	10	0.015 ± 0.001	0.048 ± 0.002	0.79 ± 0.015	0.22 ± 0.011	72.3	76.7	79.0	79.0	1.4	1.4	1.4	1.4
2.5 × 10^−3^	10	0.007 ± 0.0004	0.016 ± 0.001	0.026 ± 0.001	0.045 ± 0.002	86.8	90.1	93.09	96.9	2.1	1.8	2.2	2.1

Effect of temperature is often used to determine whether an inhibitor is physically or chemically adsorbed on a metal surface to inhibit corrosion. An enhancement in inhibition efficiency with rise in electrolyte temperature is often associated with chemisorption phenomenon while the reverse signifies physisorption. To assess the effect of temperature on the corrosion inhibition of mild steel without and with Glu(12)-2-Glu(12) and Glu(12)-2-Glu(12) + KI, weight loss experiments were done at 30, 40, 50 and 60 °C using different concentration of Glu(12)-2-Glu(12). Inspection of Table 2 reveals that inhibition efficiencies increased with increase in solution temperature, confirming the ability of surfactants to inhibit corrosion of mild steel in NaCl solution at low and relatively high temperatures and supports chemisorption^47^, which is more favoured at higher temperature because of lesser kinetic energy barrier. Further, at high temperatures, desorption of water molecules from the surface of steel is more, resulting in the larger surface area available for the adsorption of Glu(12)-2-Glu(12) gemini surfactant molecules. Also, an increase in the temperature of aqueous gemini surfactant solution increases the adsorption free energy thereby increasing the rate of adsorption on the MS surface^48^.

### Synergistic effect of KI

To observe the effect of KI on the corrosion inhibition behaviour of Glu(12)-2-Glu(12), the corrosion of mild steel in 3.5% NaCl in absence and presence of varying concentration of Glu(12)-2-Glu(12) in combination with 10 mM of KI was separately studied in the temperature range of 30–60 °C by weight loss technique. It is clear that the mild steel corrosion is only slightly inhibited in the presence of either of a small concentration of Glu(12)-2-Glu(12) (1 × 10^−5^ mM) or KI (10 mM). But in the presence of Glu(12)-2-Glu(12) + 10 mM KI, *ν* shifted to lower values. For example, when 10 mM KI was added into the 3.5% NaCl solution containing 1 × 10^−5^ mM Glu(12)-2-Glu(12), *ν* decreased from 0.056 mg cm^−2^ h^−1^ to 0.032 mg cm^−2^ h^−1^. The greater *η*_w_,% shown in the presence of I^−^ ions may be due to its less degree of hydration as the adsorbability of anions is related to the degree of hydration and less hydrated ion is preferentially adsorbed on the metal surface. Accordingly, the *η*_w_,% increased from 69% to 86%, which could be attributed to adsorption of I^-^ion over the corroded steel surface^49^. The results suggest that there is a synergistic effect between inhibitor molecules and KI, which is judged by the synergism parameter, *S*_θ_ calculated using the following equations^50^:

12 $${S}_{\theta }=1-{\theta }_{1+2}/1-\theta {^{\prime} }_{1+2}$$

13 $${\theta }_{1+2}=({\theta }_{1}+{\theta }_{2})-({\theta }_{1}{\theta }_{2})$$

where *θ*_1_ and *θ*_2_ is the surface coverage by Glu(12)-2-Glu(12) and KI, respectively and *θ*′_1+2_ is the measured surface coverage by Glu(12)-2-Glu(12) + KI. In general, *S*_*θ*_ < 1 implies an antagonistic behaviour, whereas *S*_*θ*_ > 1 implies synergistic effect^51,52^. The calculated values of *S*_*θ*_ are more than unity implying that the increased inhibition efficiency of Glu(12)-2-Glu(12) in presence of KI is only due to the synergistic effect (Table 2).

### Kinetic considerations

**Figure 2:**
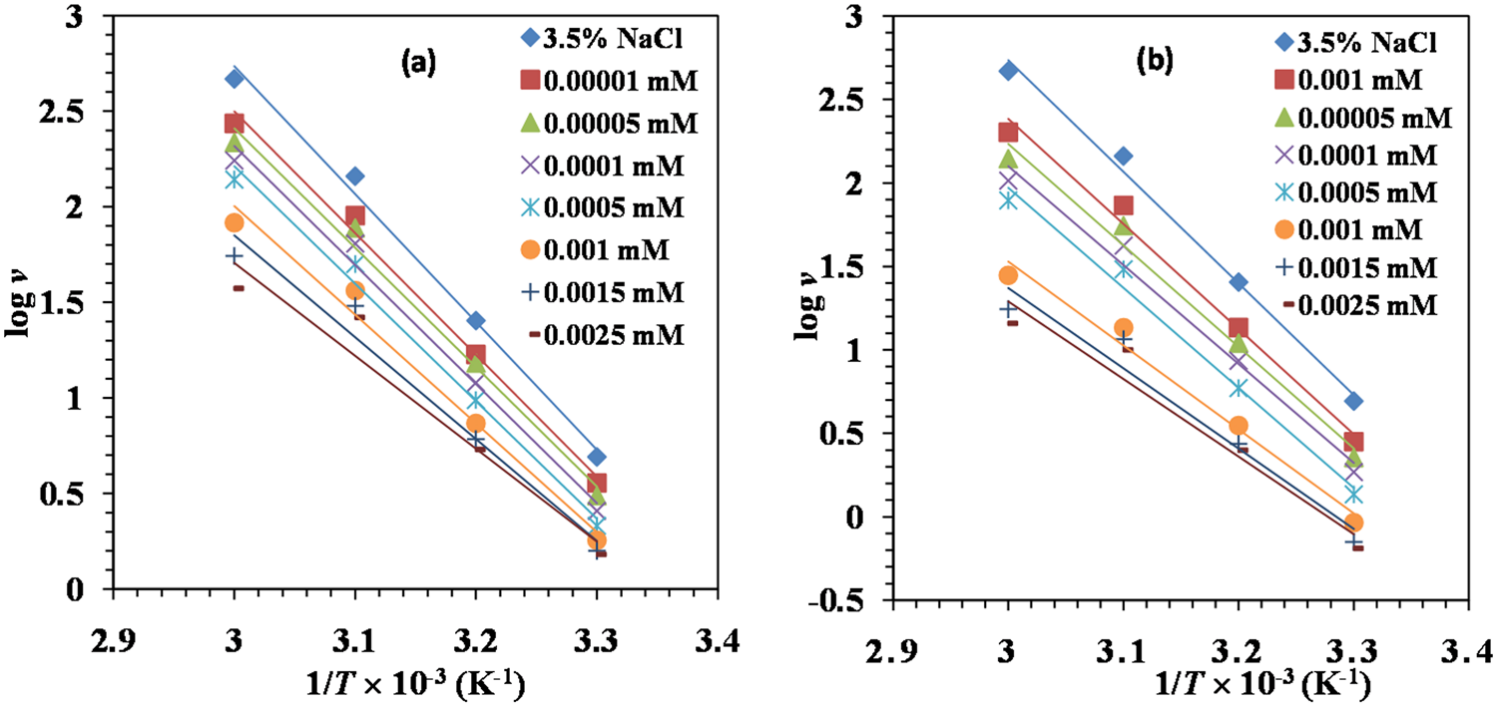
Arrhenius plots for MS in 3.5% NaCl in the absence and presence of different concentrations of (**a**) Glu(12)-2-Glu(12) and (**b**) Glu(12)-2-Glu(12) + 10 mM KI.

**Figure 3:**
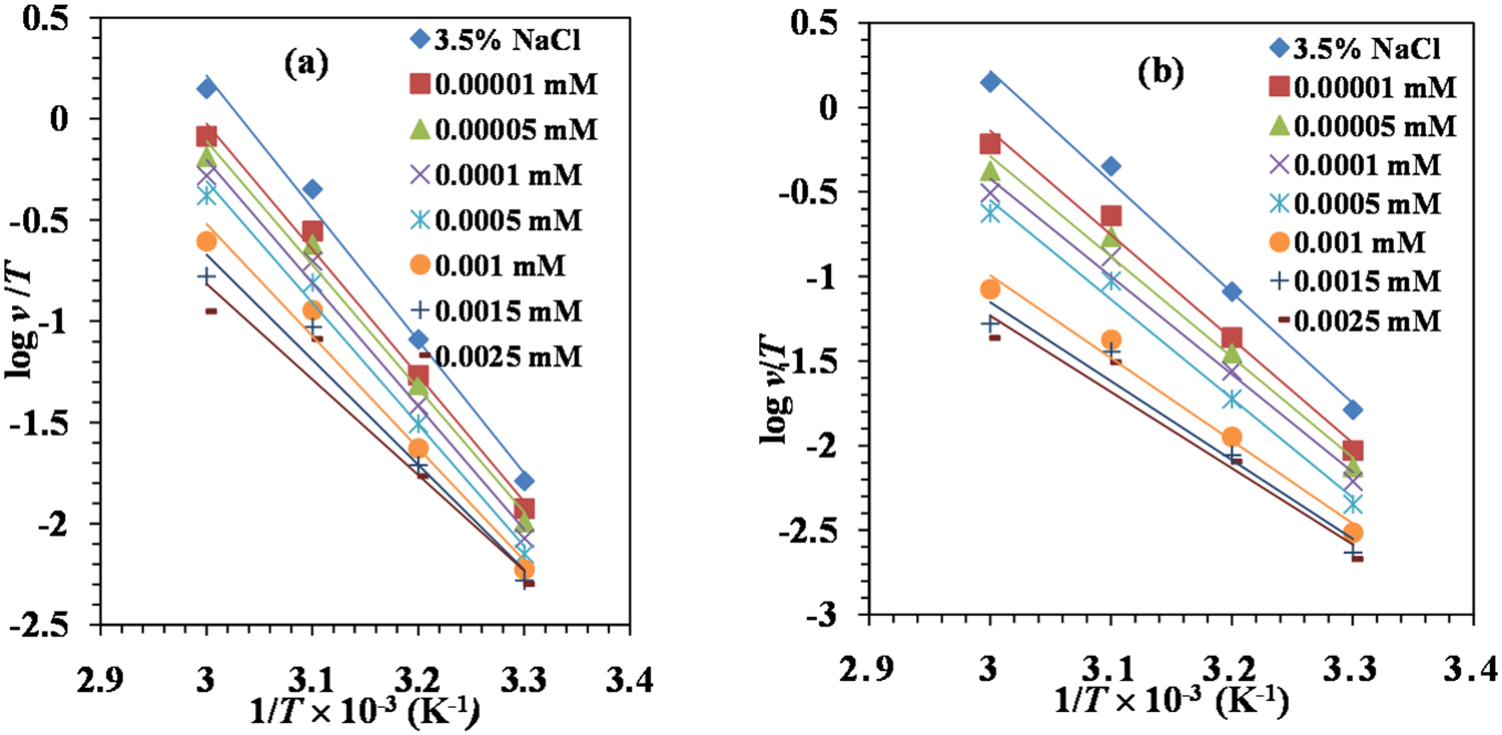
Alternative Arrhenius plots for MS in 3.5% NaCl in the absence and presence of different concentrations of Glu(12)-2-Glu(12) and Glu(12)-2-Glu(12) + 10 mM KI.

In order to further elucidate the inhibitive properties of Glu(12)-2-Glu(12) gemini surfactant and the dependence of the temperature on the corrosion rate, the activation energy, *E*_a_, enthalpy of activation, ΔH* and entropy of activation, ΔS* was calculated by Arrhenius equation and its alternative equation^52^ shown as equations (14) and (15). The relevant plots for mild steel corrosion in 3.5% NaCl in the absence and presence of various concentrations of Glu(12)-2-Glu(12) and Glu(12)-2-Glu(12) + KI at various temperatures are shown in Figs 2 and 3, respectively. The computed parameters are summarized in Table 3.

14 $$\mathrm{log}({\rm{\nu }})=\,\mathrm{log}\,A-{E}_{a}/2.303RT$$

15 $$\nu =\frac{{RT}}{{Nh}}\exp (\frac{{\rm{\Delta }}{{S}}^{\ast }}{{R}})\exp (-\frac{{\rm{\Delta }}{{H}}^{\ast }}{{RT}})$$

where ν = corrosion rate, *T* = absolute temperature, *R* = universal gas constant. *N* = Avogadro number, and *h* = Planck’s constant.

**Table 3:** Activation parameters for corrosion of MS in 3.5% NaCl containing various concentrations of Glu(12)-2-Glu(12) and Glu(12)-2-Glu(12) + 10 mM KI inhibitor.

*C* (mM)	*E*_a_ (KJ mol^−1^)	ΔH* (KJ mol^−1^)	ΔS* (KJ mol^−1^ K^−1^)
3.5% NaCl	86.45	83.83	0.065
**Glu(12)-2-Glu(12)**			
1 × 10^−5^	80.31	77.69	0.043
5 × 10^−5^	78.12	75.50	0.034
1 × 10^−4^	77.42	74.80	0.031
5 × 10^−4^	76.17	64.59	0.024
1 × 10^−3^	67.20	57.75	−0.006
1.5 × 10^−3^	60.37	57.76	−0.029
2.5 × 10^−3^	55.28	52.65	−0.047
**Glu(12)-2-Glu(12)** + **KI**			
1 × 10^−5^	79.11	76.49	0.037
5 × 10^−5^	76.15	73.53	0.025
1 × 10^−4^	71.39	68.76	0.008
5 × 10^−4^	70.41	67.78	0.002
1 × 10^−3^	55.57	52.96	−0.050
1.5 × 10^−3^	52.03	49.42	−0.063
2.5 × 10^−3^	49.14	46.44	−0.074

The lower values of *E*_a_ were observed for the inhibited systems than that for the uninhibited system indicating the chemical adsorption mechanism^53,54^. The positive value of ΔH* implies that the adsorption is endothermic reaction, which means it is good for adsorption when temperature is rising. The value of ΔH* is lower than *E*_a_, which means inhibitor has formed a stable layer on the steel surface^55^. These results reflect that Glu(12)-2-Glu(12) is a good corrosion inhibitor. The negative value of ΔS* for Glu(12)-2-Glu(12) can be explained in the following way: before the inhibitor molecules were adsorbed on the surface of mild steel, the molecules were scattered in the solution. However, as adsorption progressed the inhibitor molecules were orderly adsorbed on the surface, which led to a decrease in entropy^56^.

### Adsorption and thermodynamic considerations

**Figure 4:**
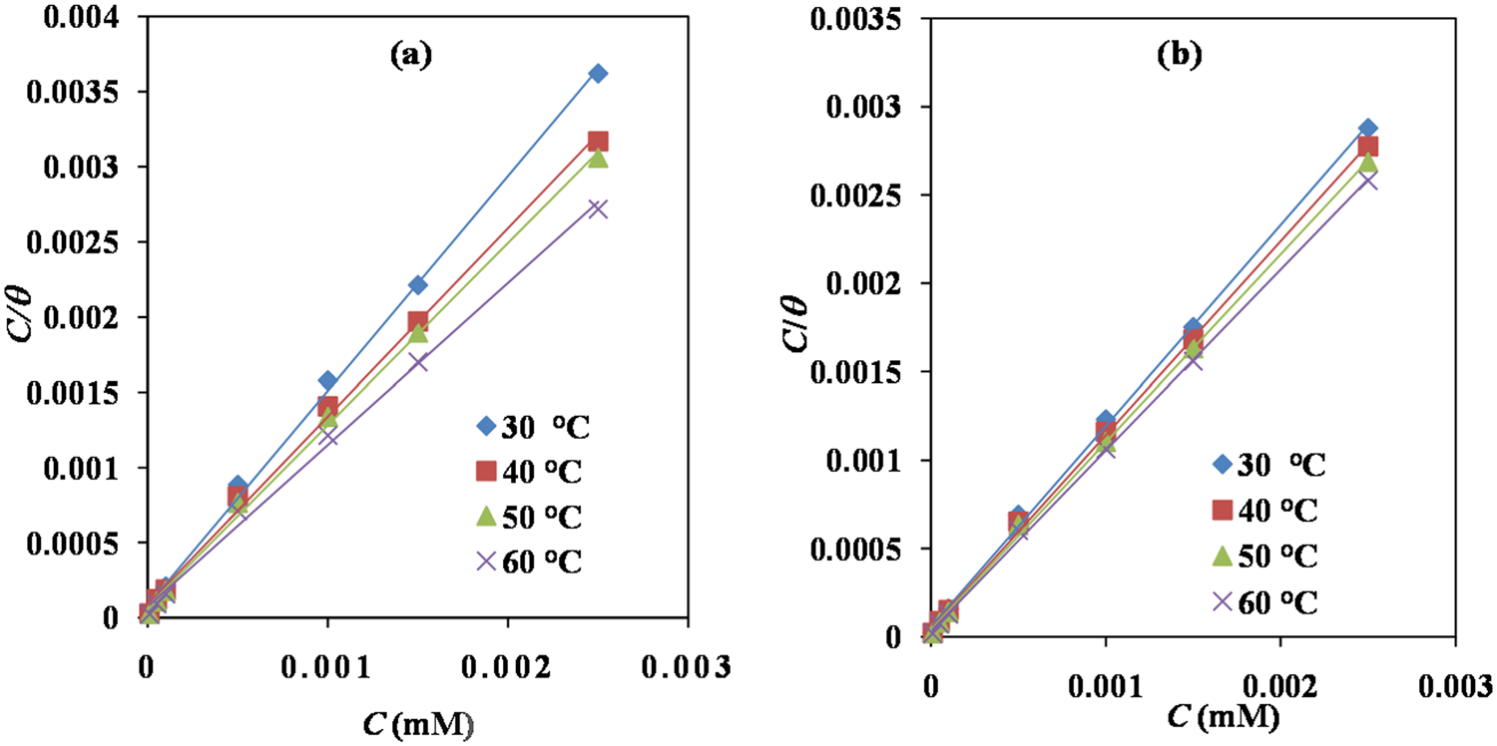
Langmuir adsorption isotherm plots for MS in 3.5% NaCl solution containing various concentrations of (**a**) Glu(12)-2-Glu(12) (**b**) Glu(12)-2-Glu(12) + 10 mM KI at 30–60 °C.

The values of surface coverage, *θ*, computed from weight loss measurements were tested graphically by fitting to various adsorption isotherms models including Langmuir, Temkin, Frumkin and Freundlich (Figs 4 and S2 (Supplementary Information)). The value of slopes and regression coefficient derived for different adsorption isotherms are given in Table S1 (Supplementary Information). The best adsorption isotherm in the present study was chosen based on the value of regression coefficient (*R*^2^) for each tested isotherm. By far, the experimental data (*R*^2^ were most close to one) for Glu(12)-2-Glu(12) or Glu(12)-2-Glu(12) + KI, best fitted the Langmuir isotherm (Fig. 4). The values of *R*^2^ were used to determine the fitting of the experimental data to this unique isotherm. The isotherm is characterized by the mathematical model given below:

16 $$C/\theta =1/{K}_{{\rm{ads}}}+C$$

where, *C* = surfactant concentration, and *K*_ads_ = adsorption equilibrium constant that can be determined from the intercept of the straight line on the *C/θ* vs. *C* plot.

Linear plots were obtained for the different systems studied indicating that the experimental results relating the adsorption of Glu(12)-2-Glu(12) on mild steel can be approximated by Langmuir adsorption isotherm. The adsorption parameters derived from the plots are listed in Table 4. The *K*_ads_ (Table 4) values are high, suggesting the strong adsorption ability of Glu(12)-2-Glu(12) on the mild steel surface. The values of *K*_ads_ (Table 4) also indicate that the binding power of the inhibitor to the metal surface increases with rise in temperature.

**Table 4:** Thermodynamic parameters of adsorption for MS in 3.5% NaCl at 30–60 °C.

*T* (°C)	*K*_ads_ (mM^−1^)	${\boldsymbol{\Delta }}{{\boldsymbol{G}}}_{{\bf{ads}}}^{{\boldsymbol{^\circ }}}$(KJ mol^−1^)	${\boldsymbol{\Delta }}{{\boldsymbol{H}}}_{{\bf{ads}}}^{{\boldsymbol{^\circ }}}$ (KJ mol^−1^)	Δ*S*^°^_ads_(J mol^−1^ K^−1^)
**Glu(12)-2-Glu(12)**				
30	11111.11	−33.58	3.37	−0.121
40	11428.57	−34.77	3.37	−0.111
50	12048.19	−36.02	3.37	−0.111
60	12500.00	−37.23	3.37	−0.112
**Glu(12)-2-Glu(12) + KI**				
30	20000.00	−35.06	13.72	−0.161
40	22222.22	−36.49	13.72	−0.116
50	25000.00	−37.97	13.72	−0.117
60	33333.33	−39.95	13.72	−0.119

The equilibrium constant of adsorption *K*_ads_ is related to the standard free energy of adsorption, ${\rm{\Delta }}{G}_{{\rm{ads}}}^{^\circ }$ with the following equation:

17 $${K}_{{\rm{ads}}}=(1/55.5)\exp (-{\rm{\Delta }}{{G}^{^\circ }}_{{\rm{ads}}}/RT)$$

where, *R* = universal gas constant and *T* = absolute temperature. The 55.5 value is the molar concentration of water in solution.

It has been demonstrated previously that higher the value of *K*_ads_, the more easily is the inhibitor adsorbed on the metal surface^57^, which means inhibitor has better inhibition performance. It can be seen from Table 4 that Glu(12)-2-Glu(12) + KI has the higher value of *K*_ads_ than Glu(12)-2-Glu(12) and thus Glu(12)-2-Glu(12) + KI out performs Glu(12)-2-Glu(12) in inhibiting performance. The values of ${\rm{\Delta }}{G}_{{\rm{ads}}}^{^\circ }$ in the range of −20–40 KJ mol^−1^ are assigned for the physical mode of adsorption. Meanwhile, ${\rm{\Delta }}{G}_{{\rm{ads}}}^{^\circ }$ values >−40 KJ mol^−1^ are assigned for the chemical mode of adsorption^58^. The values of ${\rm{\Delta }}{G}_{{\rm{ads}}}^{^\circ }$, in the present investigation, range between −33.58 to −37.23 and −35.06 to −39.95 for Glu(12)-2-Glu(12) and Glu(12)-2-Glu(12) + KI, respectively, in the temperature range 30–60 °C, indicating that the process of adsorption of Glu(12)-2-Glu(12) onto the steel surface involve mixed physisorption and chemisorption with predominantly chemisorption mechanism.

Thermodynamic models are very useful tools in explaining the adsorption mechanism of inhibitor molecules onto a metal surface. Therefore, the heat of adsorption (${\rm{\Delta }}{{H}}_{{\rm{ads}}}^{^\circ }$) was assessed using the Van’t Hoff equation:

18 $$\mathrm{log}\,{K}_{ads}=\frac{-{\rm{\Delta }}{{H}^{o}}_{ads}}{2.303RT}+{constant}$$

where, ${\rm{\Delta }}{{H}}_{{\rm{ads}}}^{^\circ }$ and *K*_ads_ are the heat of adsorption and equilibrium constant of adsorption, respectively. Figure S3(a) (Supporting Information) shows the relationship between log *K*_ads_ and 1/*T* for Glu(12)-2-Glu(12) and the absorptive heat (${\rm{\Delta }}{{H}}_{{\rm{ads}}}^{^\circ }$) was approximated from the slope of the graph under the experimental conditions and presented in Table 4. The positive value of ${\rm{\Delta }}{{H}}_{{\rm{ads}}}^{^\circ }$, displays the endothermic nature of the adsorption of the inhibitor molecules on the mild steel surface. Generally the endothermic process is attributable unequivocally to chemisorption^59^.

Another thermodynamic parameter, entropy of adsorption (${\rm{\Delta }}{S}_{{\rm{ads}}}^{^\circ }$) was calculated using basic thermodynamic equation:

19 $${\rm{\Delta }}{{G}^{o}}_{ads}={\rm{\Delta }}{{H}^{o}}_{ads}-T{\rm{\Delta }}{{S}^{o}}_{ads}$$

Values of ${\rm{\Delta }}{S}_{{\rm{ads}}}^{^\circ }$ ranges between −0.12–0.11 KJ mol^−1^ (Table 4) were obtained by substitution of corresponding values of other thermodynamic values at 30–60 °C. The negative value of ${\rm{\Delta }}{S}_{{\rm{ads}}}^{^\circ }$ for Glu(12)-2-Glu(12) can be explained by the following way: before adsorbing on the metal surface, the inhibitor molecules were in scattered manner in the solution, but in the process of adsorption, inhibitor molecules were orderly adsorbed on the mild steel surface, which led to decrease in entropy^28,59^.

## Electrochemical Study

### Open circuit potential

**Figure 5:**
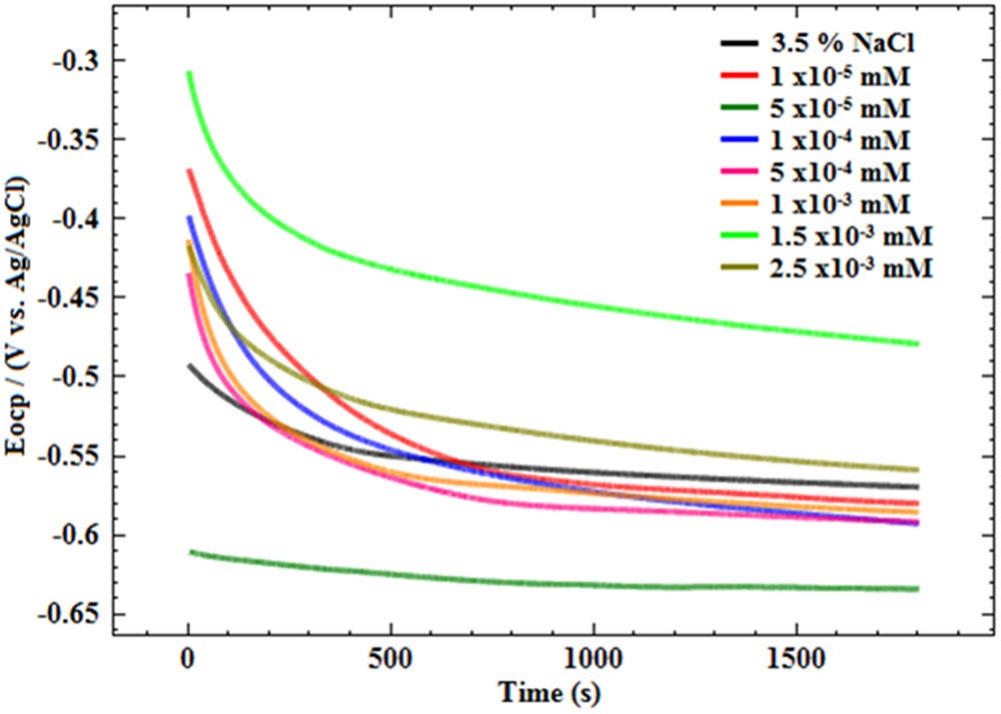
Variation of *E*_OCP_-time curves for MS in uninhibited and inhibited 3.5% NaCl solution (temperature 30 ± 2 °C).

As shown in Fig. 5, the OCP curves have similar shapes and the approximate variation trend. *E*_OCP_ changes to a positive direction at initial 320 s immersions, and then reaches a steady state after 500 s immersion. Without inhibitor, the mild steel had an *E*_OCP_ about −0.57 V vs. Ag/AgCl. For 1 × 10^−5^ mM Glu(12)-2-Glu(12), the value of *E*_OCP_ was changed to −0.59 V vs. Ag/AgCl. The negative shift in *E*_OCP_ values indicated that the Glu(12)-2-Glu(12) molecules retarded mainly the cathodic corrosion process of mild steel corrosion in 3.5% NaCl solution. The similar curves were obtained for Glu(12)-2-Glu(12) + KI (not shown here).

### Potentiodynamic polarization measurement

**Figure 6:**
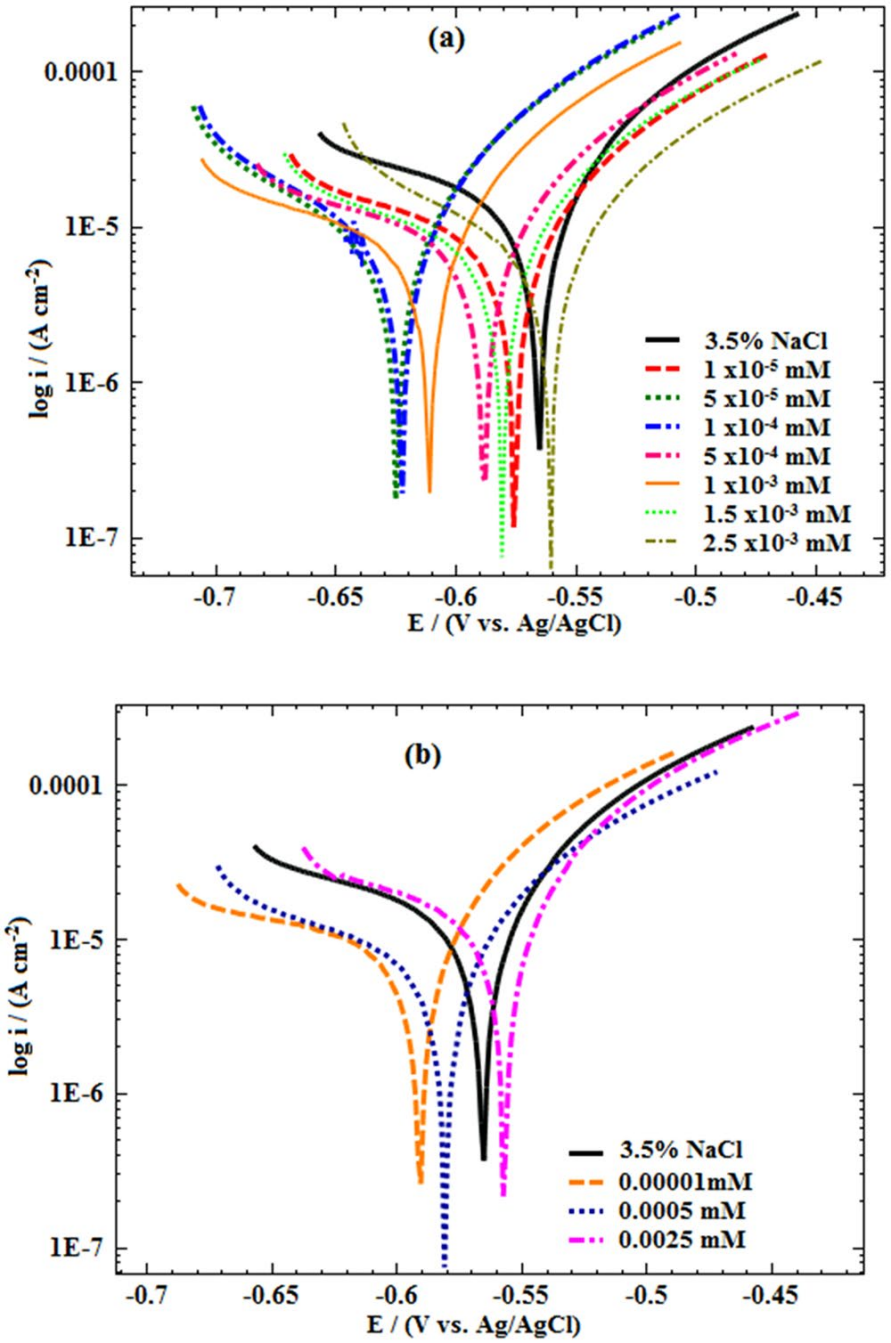
Potentiodynamic polarization curves of MS in 3.5% NaCl solution without and with different concentrations of (**a**) Glu(12)-2-Glu(12) (**b**) Glu(12)-2-Glu(12) + 10 mM KI.

Figure 6(a,b) shows potentiodynamic polarization curves for mild steel in 3.5% NaCl, both in the presence and absence of Glu(12)-2-Glu(12) and Glu(12)-2-Glu(12) + 10 mM KI at a range of concentrations. Various polarization parameters including corrosion potential (*E*_corr_), anodic (*β*_a_) and cathodic (*β*_c_) Tafel slopes, corrosion current density (*i*_corr_), and the calculated inhibition efficiency (*η*_i_) values are given in Table 5.

**Table 5:** PDP Parameters for MS in 3.5% NaCl in the absence and presence of different concentrations of Glu(12)-2-Glu(12) and Glu(12)-2-Glu(12) + 10 mM KI at 30 °C.

*C* (mM)	*E*_corr_ (V vs. Ag/AgCl)	*β*_a_(V dec^−1^)	*β*_c_(V dec^−1^)	*i*_corr_ × 10^−4^ (A cm^−2^)	*η*_i_ (%)
3.5% NaCl	−0.566	0.305	0.073	0.21 ± 0.02	—
**Glu(12)-2-Glu(12)**					
1 × 10^−5^	−0.576	0.252	0.107	0.16 ± 0.005	23.6
5 × 10^−5^	−0.624	0.298	0.068	0.12 ± 0.007	40.0
1 × 10^−4^	−0.623	0.193	0.062	0.11 ± 0.004	45.3
5 × 10^−4^	−0.589	0.151	0.056	0.09 ± 0.004	60.2
1 × 10^−3^	−0.611	0.080	0.070	0.08 ± 0.005	62.7
1.5 × 10^−3^	−0.580	0.117	0.053	0.072 ± 0.001	65.6
2.5 × 10^−3^	−0.508	0.330	0.248	0.04 ± 0.003	79.5
**Glu(12)-2-Glu(12)** + **KI**					
1 × 10^−5^	−0.604	0.279	0.054	0.098 ± 0.002	53.2
5 × 10^−4^	−0.581	0.117	0.053	0.07 ± 0.002	66.6
2.5 × 10^−3^	−0.557	0.021	0.019	0.043 ± 0.003	83.1

The variation in the values of *β*_c_ and *β*_a_ in the presence of Glu(12)-2-Glu(12) and Glu(12)-2-Glu(12) + 10 mM KI indicate that both the anodic metal dissolution and cathodic hydrogen evaluation processes are inhibited. From Table 5, it is also observed that *β*_a_ and *β*_c_ values of the inhibited acid solutions are smaller compared to that of the uninhibited solution, but does not follow a specific pattern with increase in inhibitor concentration. This suggests that the corrosion reaction mechanism is not modified^60^ due to the presence of Glu(12)-2-Glu(12). It is reported in the literature that, if the displacement in *E*_corr_ is more than 85 mV with respect to *E*_corr_ of the blank, the inhibitor can be classified as cathodic or anodic type otherwise it is of mixed type inhibitor^61^. From Table 5, it can be seen that the addition of Glu(12)-2-Glu(12) causes shift in the values of *E*_corr_ of the inhibited systems in relation to the uninhibited solution is <85 mV qualifying it as mixed type inhibitor. The value of *i*_corr_ decreases with increasing Glu(12)-2-Glu(12) concentrations, which is further decreased in presence of KI additive. For instance, the presence of 2.5 × 10^−3^ mM of Glu(12)-2-Glu(12) caused a decrease in *i*_*c*orr_ from 0.21 ± 0.02 × 10^−4^ A.cm^−2^ (for 3.5% NaCl solution) to 0.04 ± 0.003 × 10^−4^ A.cm^−2^, which further decreased to 0.036 ± 0.003 × 10^−4^ A.cm^−2^ in presence of 10 mM KI. The decrease in *i*_corr_ values is due to the adsorption of the inhibitor molecules, which blocked the active sites on the mild steel surface. Thus, the free surface area for H^+^ ions reduction was decreased while the actual reaction mechanism remains unaffected^62^.

### Electrochemical impedance spectroscopy

**Figure 7:**
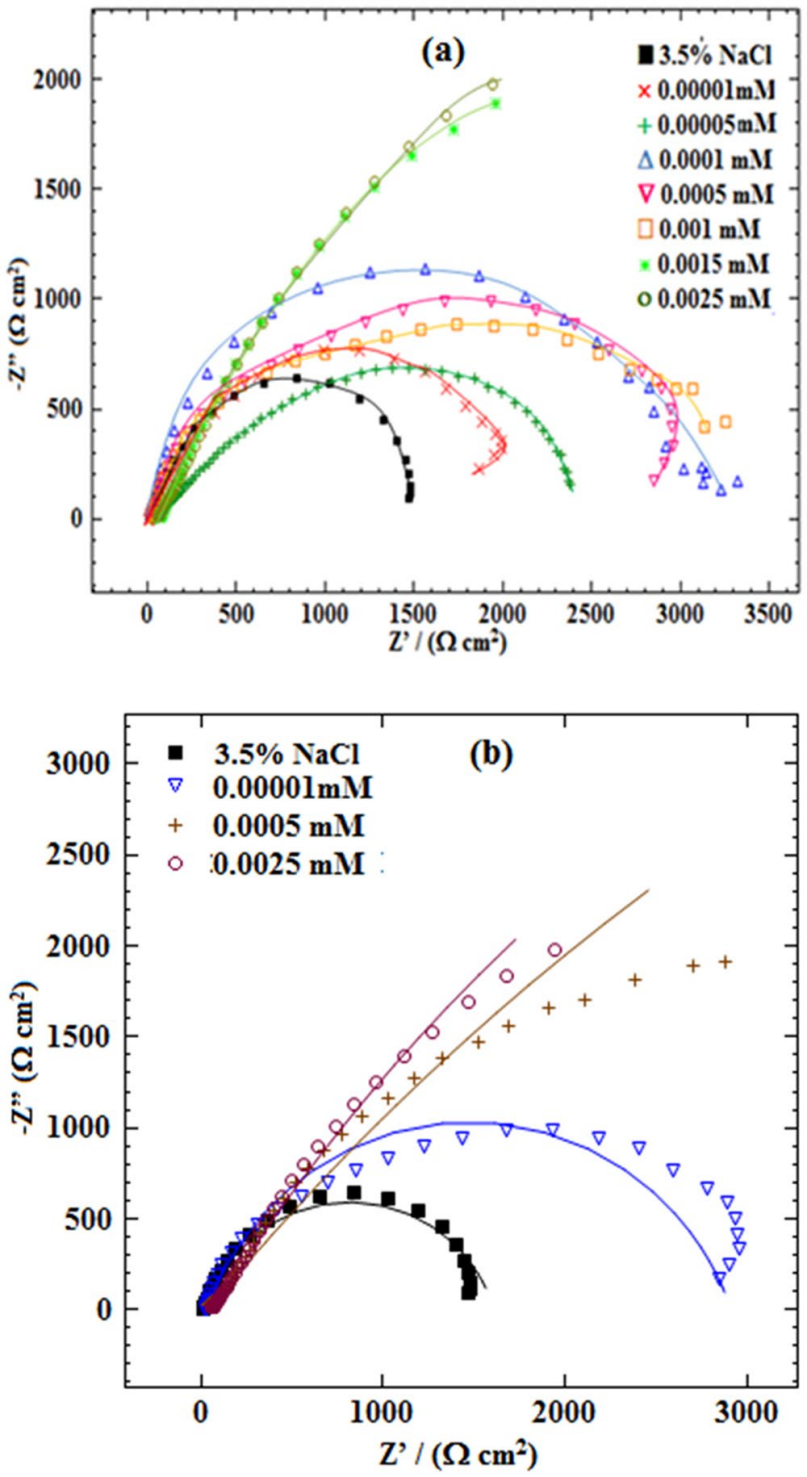
Nyquist plots of MS in 3.5% NaCl solution without and with different concentrations of (**a**) Glu(12)-2-Glu(12) (**b**) Glu(12)-2-Glu(12) + 10 mM KI.

**Figure 8:**
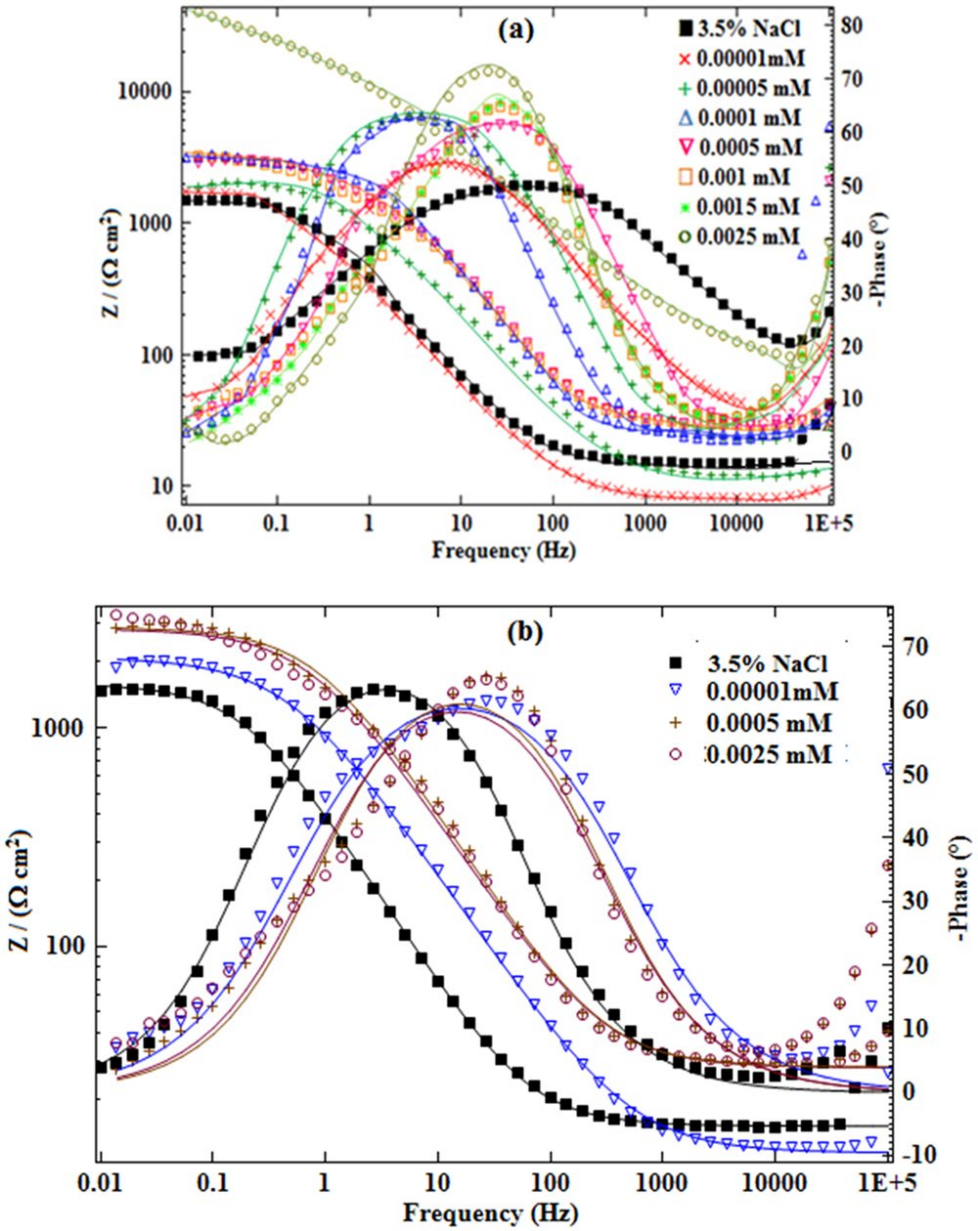
Bode plots of MS in 3.5% NaCl solution without and with different concentrations of inhibitors (**a**) Glu(12)-2-Glu(12) (**b**) Glu(12)-2-Glu(12) + 10 mM KI.

Figures 7 and 8, shows Nyquist and Bode modulus plots for mild steel corrosion in 3.5% NaCl solution without and with various concentrations of Glu(12)-2-Glu(12) and Glu(12)-2-Glu(12) + 10 mM KI at 30 °C. The impedance parameters are shown in Table 6. In the presence of Glu(12)-2-Glu(12) and Glu(12)-2-Glu(12) + 10 mM KI, the profile of impedance diagrams, which are almost semi-circle does not alter. This implies that in the investigated environment the corrosion of mild steel is mainly controlled by a charge transfer process^63^. The deviation from perfect circular shape is associated with heterogeneity of the working electrode and presence of micro roughness^64^. The inhibitor, Glu(12)-2-Glu(12) or Glu(12)-2-Glu(12) + 10 mM KI inhibited the mild steel corrosion in 3.5% NaCl solution is evident from the fact that compared with uninhibited system the semicircles of Nyquist diagrams of inhibited systems are larger in size. The above effect, which is a function of inhibitor concentration, is also evident from Bode modulus and phase angle plots where in comparison to 3.5% NaCl solution the impedance and phase angles have shifted toward greater values in inhibitor containing solutions.

**Table 6:** EIS parameters and inhibition efficiency of MS in 3.5% NaCl in absence and presence of varying concentrations of Glu(12)-2-Glu(12) and Glu(12)-2-Glu(12) + 10 mM KI at 30 °C.

*C* (mM)	*R*_s_ (Ω cm^2^)	*R*_ct_ (Ω cm^2^)	*χ*^2^ ×10^−3^	*CPE*		*C*_dl_×10^−4^ (F cm^−2^)	*η*_R_ (%)
				*Y*_0_×10^−4^ (Ω^−1^s^n^ cm^−2^)	*n*		
3.5% NaCl	18.77	1541 ± 60.1	2.3	4.85	0.9877	4.83	—
**Glu(12)-2-Glu(12)**							
1 × 10^−5^	11.37	2138 ± 87.6	2.5	1.64	0.9914	1.62	27.9
5 × 10^−5^	85.13	2573 ± 23.1	1.5	1.04	0.9929	1.03	40.1
1 × 10^−4^	65.98	3176 ± 60.3	2.7	0.47	0.9964	0.47	51.4
5 × 10^−4^	68.44	3516 ± 101.3	5.7	0.43	0.9979	0.43	56.1
1 × 10^−3^	52.97	4037 ± 48.0	2.1	0.39	0.9981	0.39	61.8
1.5 × 10^−3^	37.61	4537 ± 186.0	1.9	0.30	0.9984	0.30	66.0
2.5 × 10^−3^	32.64	5709 ± 62.8	2.1	0.28	0.9988	0.27	73.0
**Glu(12)−2-Glu(12) + KI**							
1 × 10^−5^	10.36	2621 ± 33.9	2.2	1.11	0.9973	1.1	41.2
5 × 10^−4^	81.01	5636 ± 84.5	3.1	0.28	0.9982	0.28	72.6
2.5 × 10^−3^	77.77	8857 ± 203.7	1.6	0.18	0.9998	0.18	82.6

For the analysis of the impedance spectra of Glu(12)-2-Glu(12), an appropriate equivalent circuit shown in Fig. S4 (Supporting Information) was used, which consists of the parallel combination of a capacitor, *C*_dl_ and a charge transfer resistor, *R*_ct_ in series with a solution resistor *R*_s_. A good fit with this model was obtained with our experimental data (Figs 7 and 8a,b). It is observed that the fitted data match with the experimental data, with the low chi-square (*χ*^2^) value (see Table 6). In the fitted Nyquist and Bode plots, Figs 7 and 8(a,b), the symbols depicts the experimental data, while solid lines show the best fits. The pure electric models can verify mechanistic models and approves the calculation of numerical values corresponding to the physical and/or chemical properties of the electrochemical system under investigation. The proposed circuit allows the identification of both the solution resistance and charge transfer resistance. The capacitance value is affected by defects of the surface, which can be simulated via a constant phase element (*CPE*)^65^. The *CPE* is determined by a component *Y*_0_ and a coefficient *n*, where *n* quantifies different physical phenomena, like surface in-homogeneity arising from surface roughness, inhibitor adsorption, porous layer formation, etc. In this case, the capacitance is calculated by the following mathematical formulation:

20 $${{\rm{C}}}_{{\rm{dl}}}={Y}_{{\rm{0}}}{(2\pi {f}_{{\rm{\max }}})}^{n-1}$$

where, *f*_max_ represents the frequency at which the imaginary component of the impedance is maximal on the Nyquist plot.

At 2.5 × 10^−3^ mM of studied inhibitor, the addition of KI gives a *R*_ct_ value of 8857 ± 203.7 Ω cm^2^ and *C*_dl_ value of 0.18 × 10^−4^ µFcm^−2^, while Glu(12)-2-Glu(12) gives respective values of 5709 ± 62.8 Ω cm^2^ and 0.27 × 10^−4^ µFcm^−2^. Under any given conditions, *R*_ct_ Glu(12)-2-Glu(12) + KI > *R*_ct_ Glu(12)-2-Glu(12), while *C*_dl_ Glu(12)-2-Glu(12) + KI < *C*_dl_ Glu(12)-2-Glu(12), implying improved inhibition by Glu(12)-2-Glu(12) + KI than by Glu(12)-2-Glu(12). In both cases, generally, *η*_R_ increases with the concentration of the inhibitor, and follows the order: *η*_R_ Glu(12)-2-Glu(12) + KI > *η*_R_ Glu(12)-2-Glu(12).

In Fig. 8(a,b), the Bode-phase angle diagrams for mild steel with and without various concentrations of Glu(12)-2-Glu(12) and Glu(12)-2-Glu(12) + KI are shown. The increase in the absolute value of impedance |Z| at low frequencies confirms the corrosion protection offered by the studied inhibitor because when the inhibitor is added, there is an increase in the impedance value of one order of magnitude. The increase of the phase angle in the presence of the inhibitor indicates that it is adhering to the metallic surface^66^. Additionally, one time constants could clearly be observed for all concentrations.

### Surface characterization

**Figure 9:**
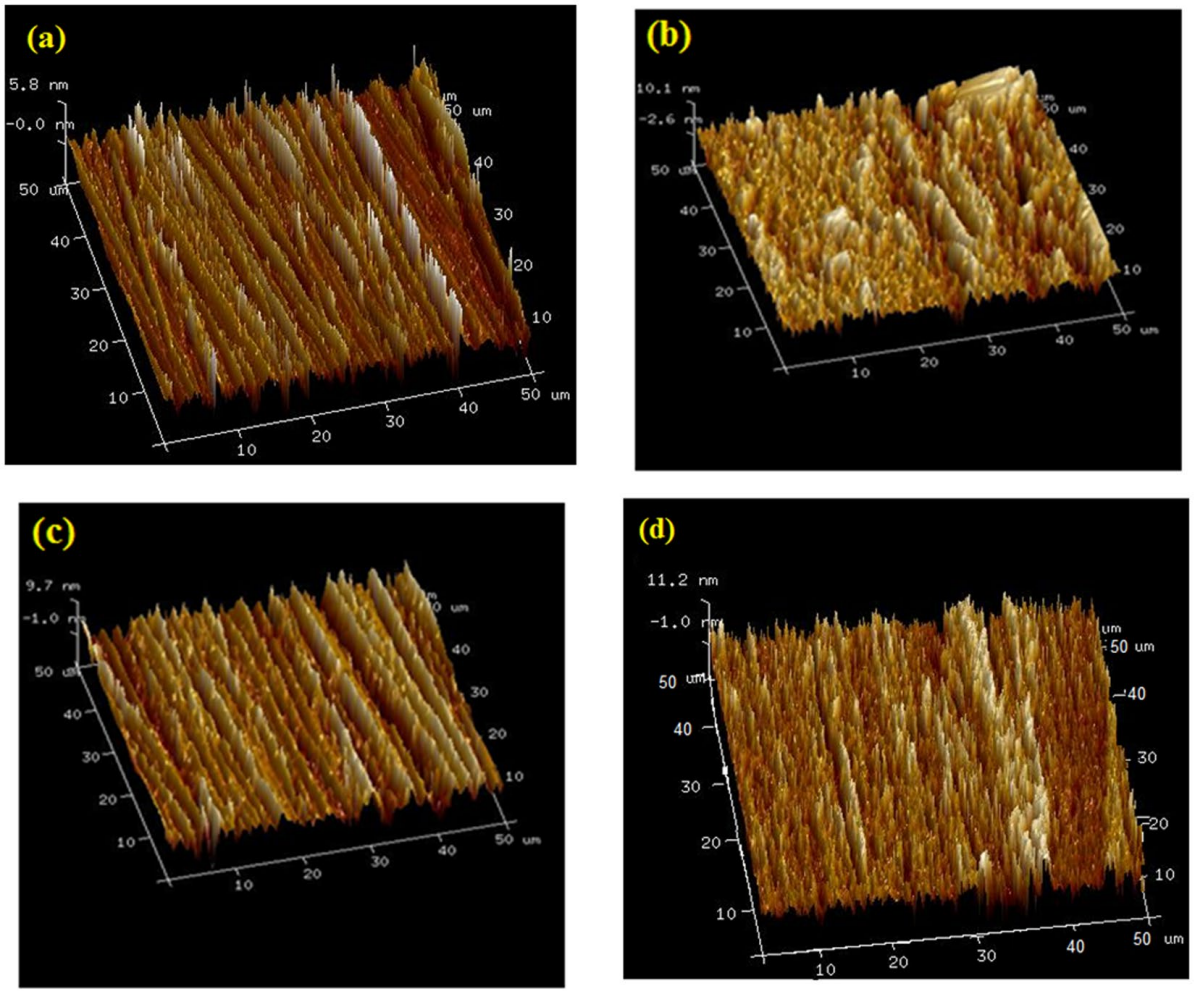
AFM images of MS after 6 h immersion: (**a**) polished MS prior to immersion, (**b**) MS in 3.5% NaCl solution, (**c**) MS in 3.5% NaCl solution with 2.5 × 10^−3^ mM Glu(12)-2-Glu(12), (**d**) in 3.5% NaCl solution with 2.5 × 10^−3^ mM Glu(12)-2-Glu(12) + 10 mM KI.

**Figure 10:**
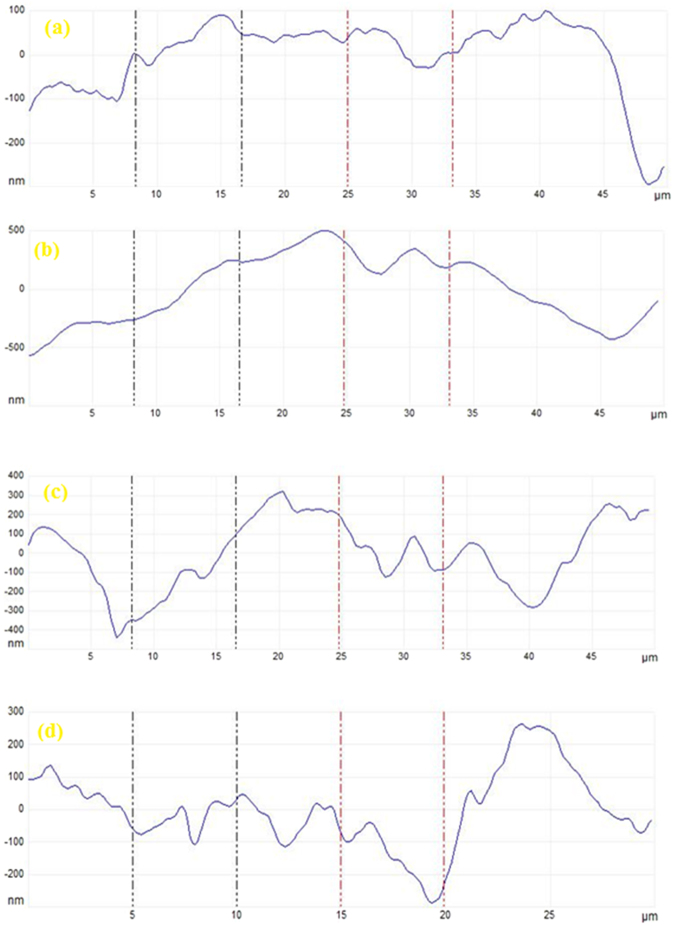
Height profile images of the MS surface (**a**) polished MS prior to immersion (**b**) MS in 3.5% NaCl solution (**c**) MS in 3.5% NaCl solution with 2.5 × 10^−3^ mM Glu(12)-2-Glu(12 (**d**) MS in 3.5% NaCl solution with 2.5 × 10^−3^ mM Glu(12)-2-Glu(12)+10 mM KI.

AFM was used to get the 3-D topography of mild steel specimen. Analysis of the AFM results allowed the quantification of surface roughness over an area of 50 μm × 50 μm. The 3-D AFM images are shown in Fig. 9(a–d). Figure 9(a) shows the mild steel surface before to immersion, which looks smooth and uniform. The mild steel surface in the uninhibited NaCl solution has become quite rough (Fig. 9b) due to the unhindered attack of the corrosive solution. However, in the presence of optimum concentration of the Glu(12)-2-Glu(12) and Glu(12)-2-Glu(12) + 10 mM KI (Fig. 9c,d), the roughness is greatly diminished in comparison to that in the absence of inhibitor. The average surface roughness of the mild steel before immersion is 81 nm, which is raised to 447 nm after immersion in uninhibited 3.5% NaCl solution. In the presence of Glu(12)-2-Glu(12) and Glu(12)-2-Glu(12) + KI, the roughness decreased to 154 nm and 129 nm, respectively. The results are further supported by height profiles graphs (Fig. 10). In comparison to the coupon immersed in 3.5% NaCl solution, which is shown to exhibit uneven height profile, the coupons immersed in inhibited NaCl solution have smoother height profile curve.

**Figure 11:**
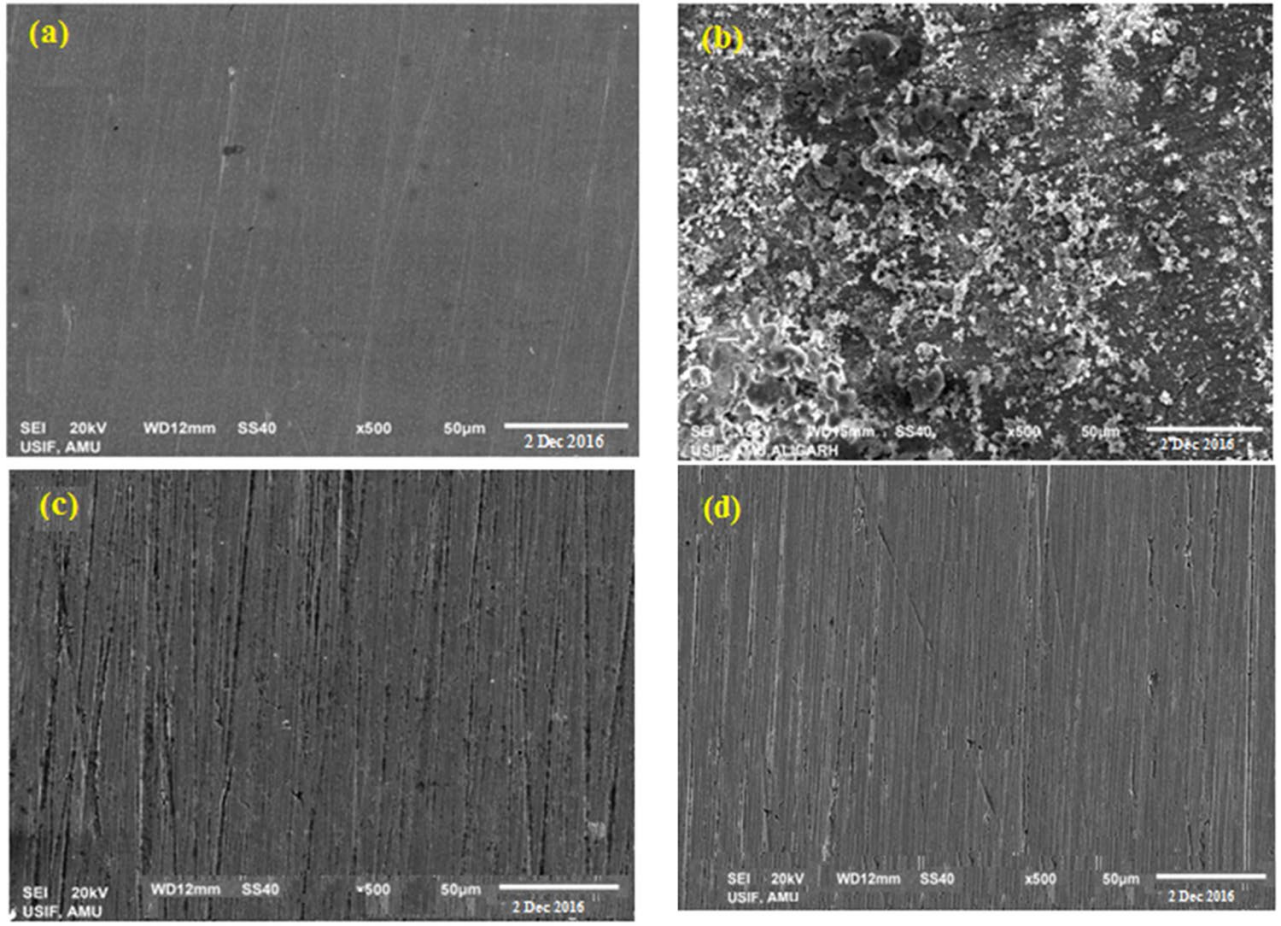
SEM images of the MS after 6 h immersion in 3.5% NaCl solution: (**a**) polished MS prior to immersion, (**b**) MS in 3.5% NaCl solution, (**c**) MS in 3.5% NaCl solution with 2.5 × 10^−3^ mM Glu(12)-2-Glu(12) (**d**) MS in 3.5% NaCl solution with 2.5 × 10^−3^ mM Glu(12)-2-Glu(12) + 10 mM KI.

The surface morphologies of freshly polished mild steel surface and mild steel surface immersed in the corrosive solutions for 6 h in the absence and presence of optimum concentration of the studied inhibitor are shown in Fig. 11(a–d). The freshly polished mild steel shows smooth, non-corroded surface with polishing scratches (see Fig. 11a). The mild steel retrieved from 3.5% NaCl without the inhibitor exhibits strongly damaged and corroded surface due to direct attack in the uninhibited corrosive environment (Fig. 11b). It is manifested from Fig. 11(c,d) that the mild steel specimens immersed in the inhibited aggressive solution are only slightly damaged compared to the one recovered from 3.5% NaCl solution. This is a sign of the existence of an inhibitive film of the Glu(12)-2-Glu(12) molecules on the steel surface thereby shielding it from direct attack. In the presence of combined Glu(12)-2-Glu(12) + KI (Fig. 10d), the steel specimen has a better morphology and smooth surface compared with that of the surface immersed in Glu(12)-2-Glu(12) solution. A comparatively lesser destruction of the mild steel surface might be due to the specific adsorption of I^−^ ions on the mild steel which facilitates the adsorption of Glu(12)-2-Glu(12) molecules.

In order to confirm the composition of corrosion products formed on the mild steel surface, the surface of specimens before and after 6 h immersion in the uninhibited and inhibited (inhibitor concentration 2.5 × 10^−3^ mM) 3.5% NaCl solution was analyzed using EDAX. The results of the EDAX analysis are shown in Fig. S5(a–d) (Supporting Information). The surface of mild steel prior to suspension in the 3.5% NaCl solution shows an intense peak of Fe along with the peaks of other elements constituting the mild steel (Fig. S5(a) supporting information). The EDAX spectrum obtained after the immersion of the mild steel in the 3.5% NaCl solution exhibits the additional peaks of Cl (Fig. S5(b) supporting information), which is ascribed to the free corrosion of unprotected mild steel in the 3.5% NaCl solution. Due to unhindered corrosion of mild steel the peak of Fe is highly suppressed. An additional and prominent peak of O, which was absent in Fig. S5(a) (Supporting Information), was evident due to the formation of complex oxides/hydroxides on the surface. The corrosion of mild steel has also resulted in the loss of some of the elements making the steel. In the presence of the inhibitor Glu(12)-2-Glu(12), the peak of Cl is absent and an additional peak of N (due to N atoms from the inhibitor) is present (Fig. S5(c) Supporting Information). This is associated to the absence of corrosion due to the formation of a protective adsorption film having N covering on the mild steel surface. As a result of the formation of a protective covering the intensity of the Fe peak is also enhanced whereas the intensity of O peak is lowered. In presence of Glu(12)-2-Glu(12) + KI (Fig. S5(d) Supporting Information), the extent of corrosion is further lowered and some of the peaks (e.g. Mn and Si) lost due to corrosion reappears.

### Quantum chemical calculations

**Figure 12:**
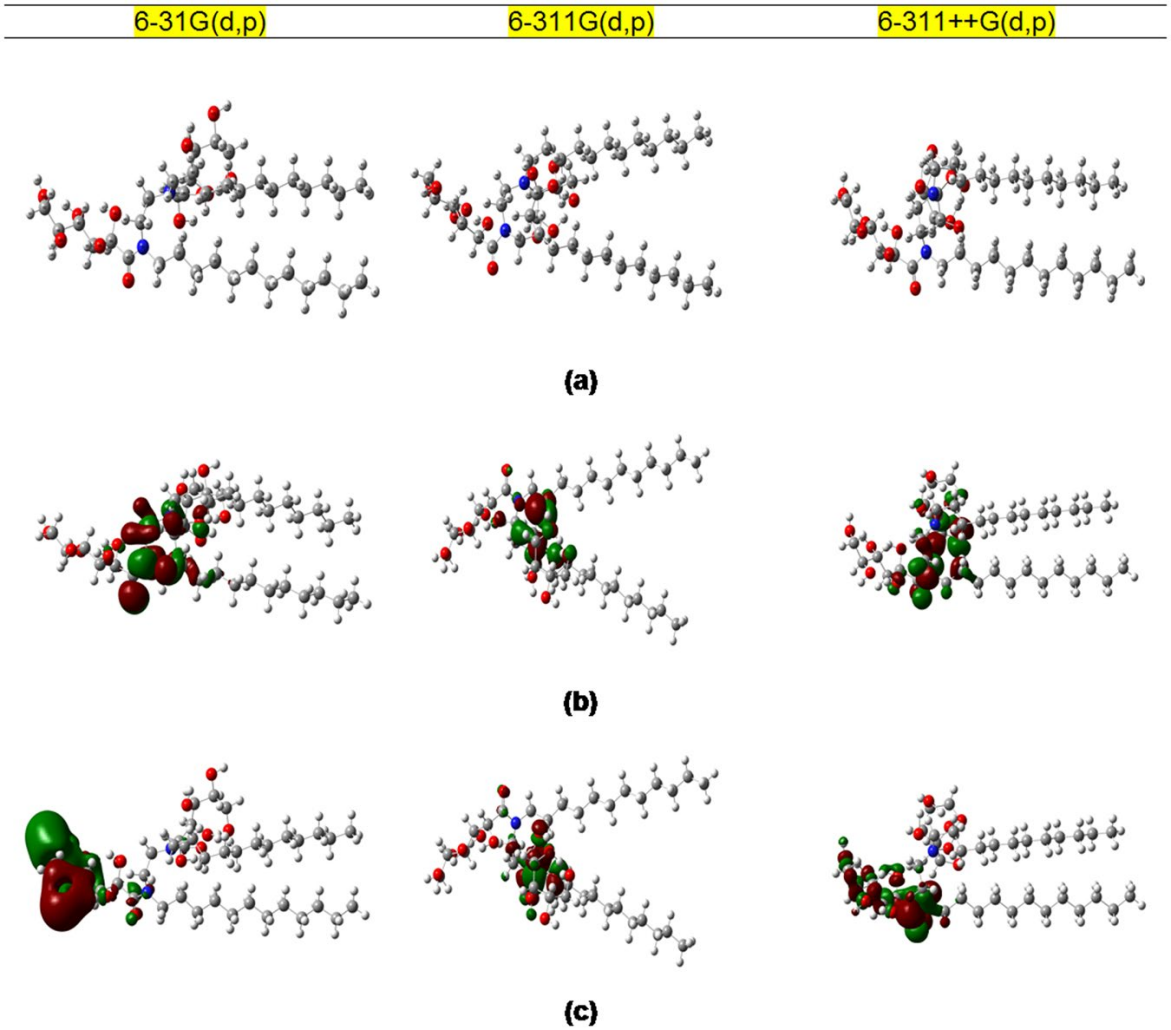
Quantum chemical results of Glu(12)-2-Glu(12) molecule obatined by DFT method at B3LYP with 6–31G (d, p), 6–311G (d, p) and 6–311G++ (d, p) basis set: (**a**) optimized molecular structure, (**b**) HOMO, (**c**) LUMO.

Quantum chemical calculations were performed to explore the relationship between electronic structure of the Glu(12)-2-Glu(12) and its inhibition ability. Optimized structures, the highest occupied molecular orbital (HOMO) and the lowest unoccupied molecular orbital (LUMO) density distribution of studied inhibitors are presented in Fig. 12(a–c). Indeed, some calculated quantum chemical indices including *E*_HOMO_, *E*_LUMO_, Δ*E*, *I*, *A*, *χ*, *η*, and Δ*N* are given in Table 7. The trends of properties obtained using different basis sets are the same (Table 7).

**Table 7:** Calculated quantum chemical parameters of the studied inhibitor using the DFT method at B3LYP with 6–31 G (d, p), 6–311 G (d, p) and 6–311 G++ (d, p) basis sets.

	6–31 G(d, p)	6–311 G(d, p)	6–311++G(d, p)
*E*_HOMO_	−3.256	−2.807	−2.227
*E*_LUMO_	3.995	3.110	1.128
Δ*E*	7.252	5.917	3.356
*I*	3.256	2.807	2.228
*A*	−3.995	−3.110	−1.128
*χ*	−3.626	−0.152	0.549
*η*	3.626	2.958	1.677
Δ*N*	0.715	0.840	1.272

According to the frontier molecular orbital theory, the formation of a transition state occurs due to interactions between the frontiers orbitals (HOMO and LUMO) of the reactants^67^. The HOMO is linked with the ability of an inhibitor molecule to give electrons and to bond to a metal surface. Higher values of *E*_HOMO_ indicate that a molecule is more likely to give electrons to appropriate acceptor molecules. In contrast, the LUMO exhibits the electron accepting ability of a molecule, with lower values of *E*_LUMO_ implying a higher electron accepting ability^68^. Glu(12)-2-Glu(12) adsorbed on the mild steel surface due to the interaction of partially filled d orbital {[Ar] 4s^2^3d^6^} of iron atoms and unshared electron pairs in nitrogen and oxygen. The HOMO of Glu(12)-2-Glu(12) could bond with the partially filled 3d orbital of iron atom while the LUMO of Glu(12)-2-Glu(12) could interact with the 4s orbital of iron atom. As the Δ*E* reflects the stability of a molecule, smaller values of Δ*E* indicate that a molecule could potentially be adsorbed more easily on a metal surface. As Δ*E* decreases, the reactivity of a molecule towards a metal surface increases, thus leading to enhancement of the inhibition efficiency of the molecule^38,69^.

The values of *E*_HOMO_ and *E*_LUMO_ of the inhibitor molecule are association with *I* and *A*, respectively^70^. The values of *I* and *A* are defined as −*E*_HOMO_ and −*E*_LUMO_, respectively and the obtained values are used to calculate *η* and *χ*.

21 $$\eta =\frac{I-A}{2}$$

22 $$\chi =\frac{I+A}{2}$$

Calculated values of *χ* are also mentioned in Table 7, which denotes the tendency of an atom to attract the shared pair of electron towards itself. The values of *η* determines both the stability and reactivity of a molecule which suggests the resistivity of the inhibitor for the physical adsorption process. Soft molecules with small energy gaps are far more reactive than hard ones with large energy gaps, as they could readily offer electrons to an acceptor.

The fraction of electrons transferred^71^ (Δ*N*) from the inhibitor to MS surface can be calculated by using *χ* and *η* values, is calculated according to equation (23).

23 $${\rm{\Delta }}N=\frac{{\chi }_{Fe}+{\chi }_{inh}}{2({\eta }_{Fe}+{\eta }_{inh})}$$

where *χ*_Fe_ and *χ*_inh_ represent the electronegativity and the *η*_Fe_ and *η*_inh_ represent the absolute hardness of iron and the inhibitor molecules, respectively. The theoretically calculated value of *χ*_Fe_ for iron metal is 7 eV mol^−1^ and the *η*_Fe_ is 0 eV mol^−1^. These values are appropriately substituted to calculate Δ*N*. Recently, several researchers^71–73^ reported that the incorporation of 7 eV mol^−1^ as the magnitude of *χ*_Fe_ is conceptually incorrect as it is only related to free electron gas Fermi energy of Fe atom in which the interactions among the electrons had not been considered. Thus work function (∅) of the metal surface instead of *χ*_Fe_, is used, as it is more appropriate measure for its electronegativity. Therefore, equation 23 is rewritten as follows.

24 $${\rm{\Delta }}N=\frac{\varphi -{\chi }_{inh}}{2({\eta }_{Fe}+{\eta }_{inh})}$$

The obtained DFT derived *ϕ* value for Fe(110) plane is 4.82 eV^72^. These values are appropriately substituted to calculate Δ*N*. Values of Δ*N* exhibit the path of the electron transfer between inhibitor and metal surface. The Δ*N* exhibit the inhibitive performance of the inhibitors resulted from electron donations. In the current investigation, Δ*N* values are greater than zero indicating electron transfer from the inhibitor to the MS surface^28^.

## MD Simulations

**Figure 13:**
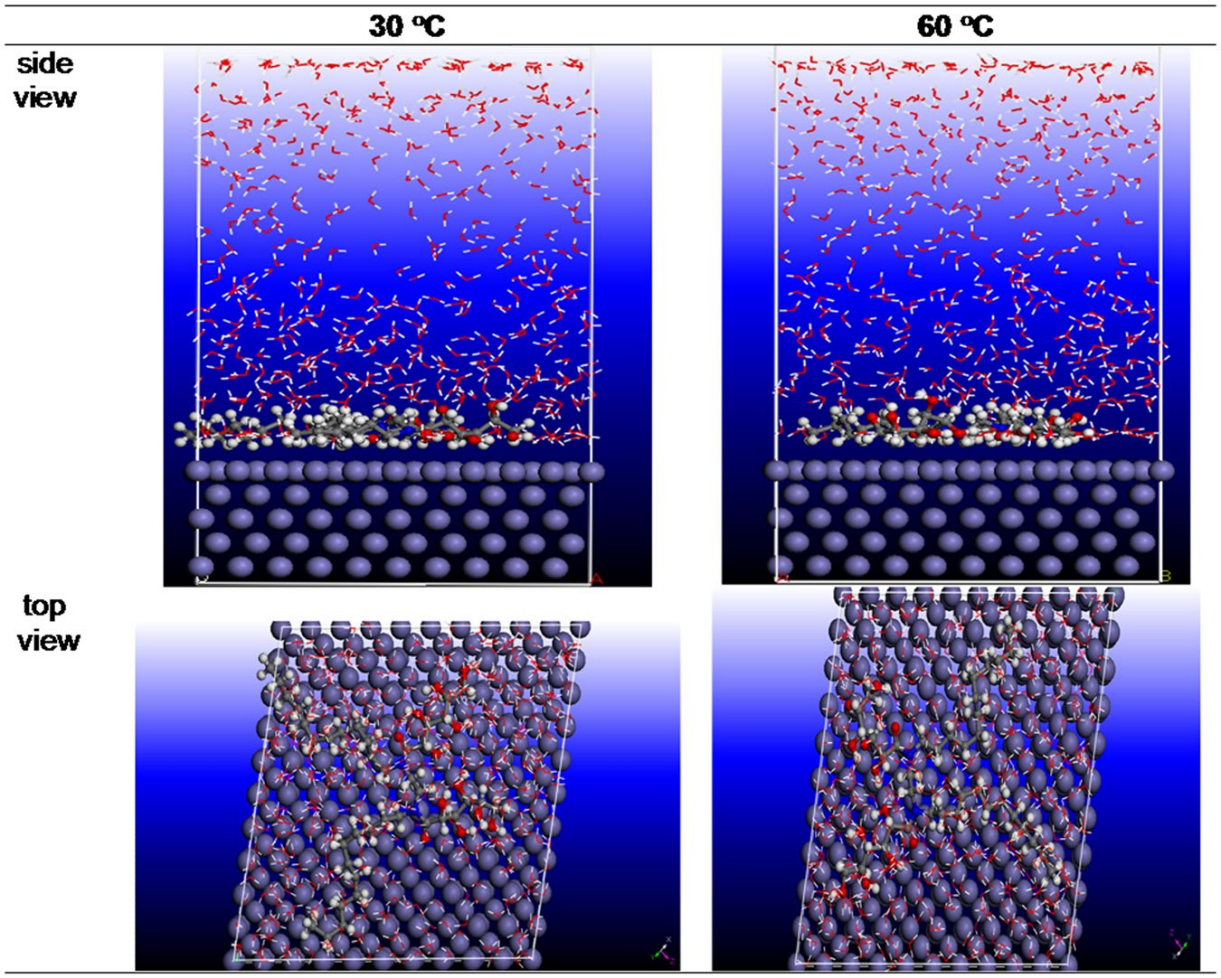
Equilibrium adsorption configurations of Glu(12)-2-Glu(12) on the Fe (110) surface at 30 and 60 °C obtained by MD simulation.

Because strong correlation exists between electronic properties of inhibitor and corrosion effectiveness, DFT calculations are very useful for the overall understanding of electronic properties of corrosion inhibitors. On the other side, understanding the interactions of inhibitor molecules with metal surface is also of great importance. In this case, MD simulations can be particularly useful for the understanding of corrosion inhibition process. In the current study, the binding and interaction energies of the adsorbed inhibitor have been approximated when the simulation system reached their equilibrium state^72^. The best adorable top and side views of adsorption configuration of the studied molecule on Fe(110) surface at 30 to 60 °C is shown in Fig. 13 while the interaction and binding energies at both temperatures are placed in Table 8. Looking at Fig. 13, what stands out is that inhibitor molecule at both temperatures had been moved in nearly parallel or flat disposition which provide larger blocking area by inhibitor molecule preventing the surface from corrosion through a formation of a barrier layer between the metal surface and the aggressive media. The large negative values of the interaction energies for both temperatures indicating that the interaction between inhibitor molecules and Fe(110) surface is spontaneous, strong and stable^74^. On the other hand, the high magnitude of the binding energies suggesting that the adsorption system is more stable and that there is more than one bond to the iron surface per inhibitor molecule^75^. The presence of many reactive sites, i.e. hydroxyl groups, nitrogen atoms as well as carbon chains in the inhibitor molecule caused highly improved inhibitive properties. Furthermore, the increase of the temperature from 30 to 60 °C increases the interactive forces of the inhibitor, in accordance with experimental results.

**Table 8:** Selected energy parameters obtained from MD simulations for adsorption of inhibitor on Fe(110) surface.

System	30 °C		60 °C	
	${{\boldsymbol{E}}}_{{\bf{interaction}}}$ (kJ/mol)	${{\boldsymbol{E}}}_{{\bf{binding}}}$ (kJ/mol)	${{\boldsymbol{E}}}_{{\bf{interaction}}}$ (kJ/mol)	${{\boldsymbol{E}}}_{{\bf{binding}}}$ (kJ/mol)
Fe (110)/**Inh**/500H_2_O	−503.77	503.77	−684.41	684.41

## Conclusions

1. The corrosion of mild steel in 3.5% NaCl has been inhibited by the addition of Glu(12)-2-Glu(12) and KI.
2. Significant improvement in inhibiting efficiency was observed in the presence of the mixture of Glu(12)-2-Glu(12) and KI. The inhibition efficiency was enhanced to 97% and showed a synergism of inhibition.
3. Glu(12)-2-Glu(12)+KI synergistic inhibitor system was found to be an excellent and cost-efficient inhibitor for mild steel corrosion in NaCl solution.
4. The values of *S*_θ_ are greater than unity showing the corrosion inhibition brought about by Glu(12)-2-Glu(12) in combination with the KI is synergistic in nature and co-operative adsorption between halides and Glu(12)-2-Glu(12) prevails over competitive adsorption.
5. According to *E*_corr_, of both the systems, Glu(12)-2-Glu(12) and Glu(12)-2-Glu(12) + KI affected both anodic and cathodic reactions so can be classified as mixed type inhibitor.
6. SEM images show that the best morphology surface is obtained for Glu(12)-2-Glu(12) + KI inhibitor.
7. AFM images showed that the molecules of Glu(12)-2-Glu(12) + KI protect the mild steel surface and prevent it from having direct contact with corrosive ions.
8. Experimental findings were adequately supported by quantum chemical calculations.
9. The configuration of molecules on metal surface was simulated using MD. The results reveal that Glu(12)-2-Glu(12) adsorbed onto the mild steel surface in a nearly parallel or flat disposition with large negative values of the interaction energies for both temperatures indicating that the interaction between inhibitor molecules and Fe(110) surface is spontaneous, strong and stable.

## Electronic supplementary material

Supporting information
