# Supplementary material for: Sugar based N,N′-didodecyl-N,N′digluconamideethylenediamine gemini surfactant as corrosion inhibitor for mild steel in 3.5% NaCl solution-effect of synergistic KI additive
Source: Sci Rep. 2018 Feb 27;8:3690. doi: 10.1038/s41598-018-21175-6 (PMC5829231; doi:10.1038/s41598-018-21175-6)
Supplement: Supplementary file 1 — Supporting information [file 41598_2018_21175_MOESM1_ESM.pdf]

Sugar based *N,N'*-didodecyl-*N,N'*digluconamideethylenediamine gemini surfactant as corrosion inhibitor for mild steel in 3.5% NaCl solution-effect of synergistic KI additive

Ruby Aslam[1], Mohammad Mobin[*1], Jeenat Aslam[1], & Hassane Lgaz[2,3]

[1]Corrosion Research Laboratory, Department of Applied Chemistry, Faculty of Engineering and Technology, Aligarh Muslim University, Aligarh 202002, India

[2]Laboratory of Applied Chemistry and Environment, ENSA, Ibn Zohr University, PO Box 1136, 80000 Agadir, Morocco

[3]Department of Applied Bioscience, College of Life & Environment Science, Konkuk University, 120 Neungdong-ro, Gwangjin-gu, Seoul 05029, South Korea.

[*]Correspondence should be addressed to (Mohammad Mobin email: drmmobin@hotmail.com)

Supporting Information

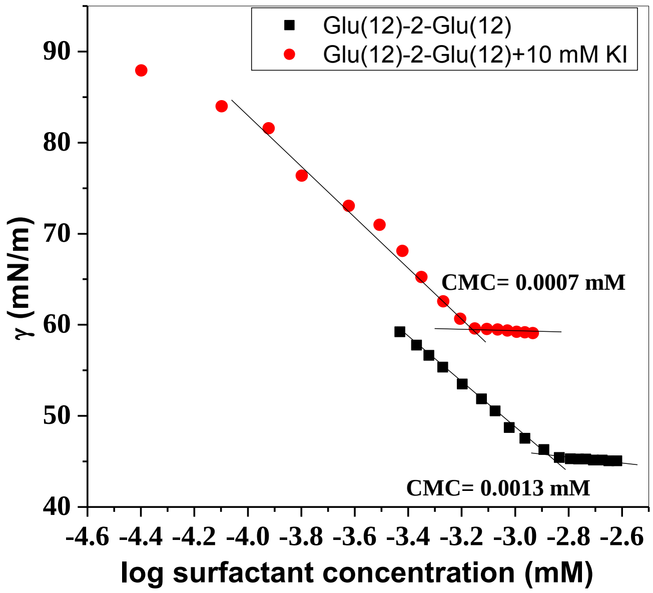

Figure S1. Variation in the surface tension as a function of concentration for Glu(12)-2-Glu(12) and Glu(12)-2-Glu(12) + 10 mM KI.

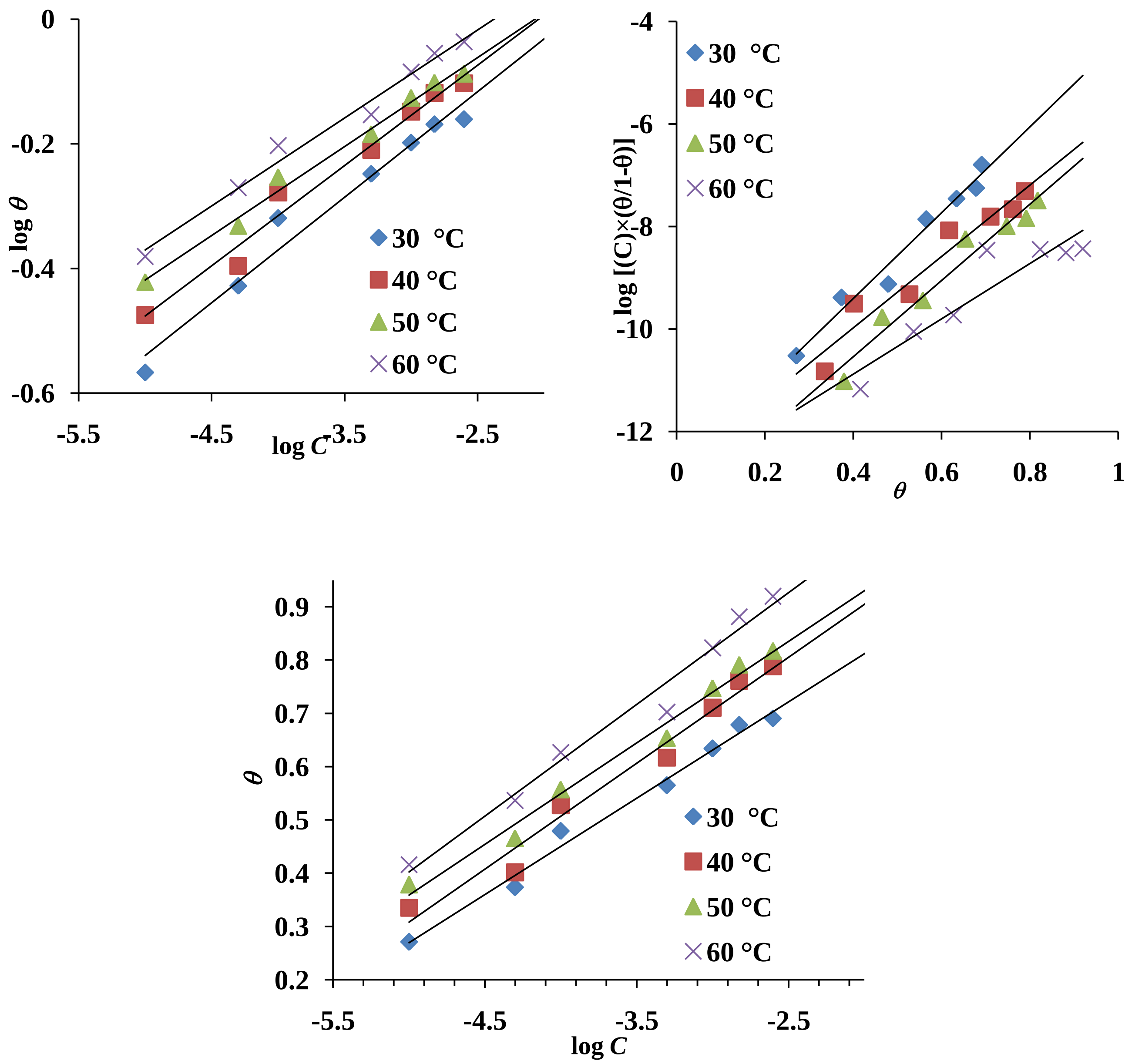

Figure S2. (a) Freundlich, (b) Frumkin, and (c) Temkin adsorption isotherm plots.

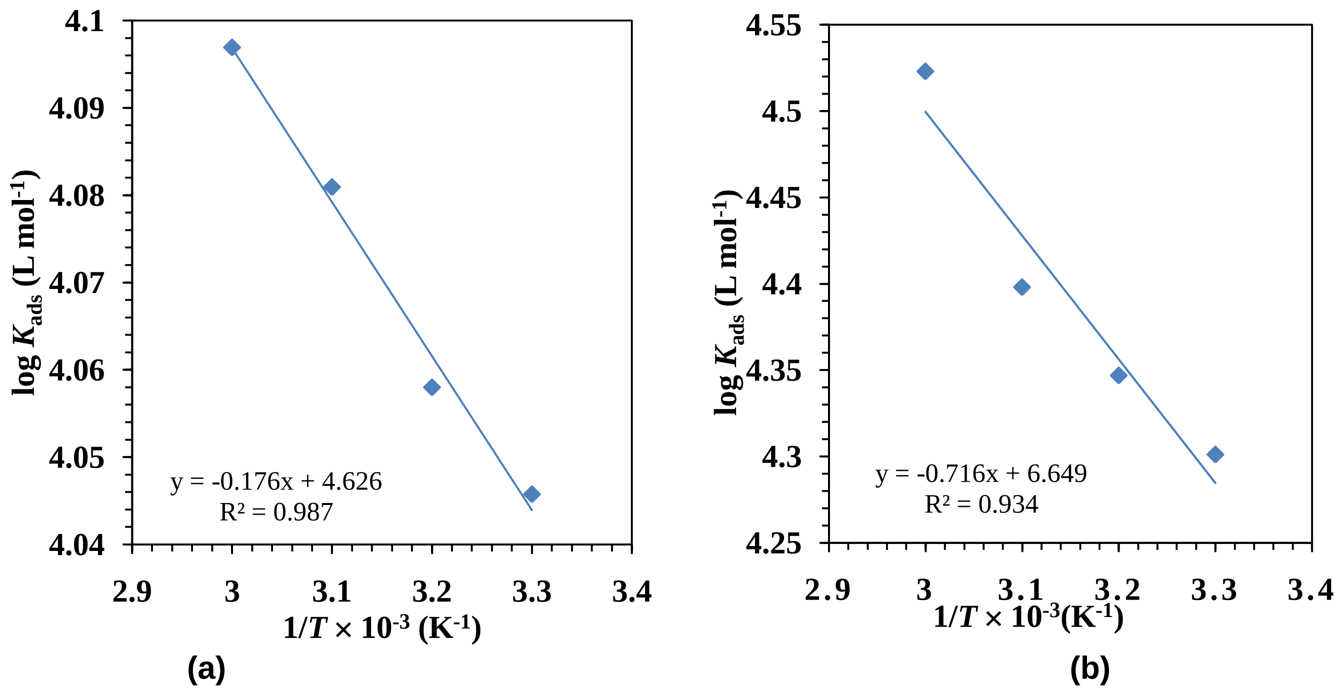

Figure S3. Linear regression between log K_{ads} and 1/T (a) Glu(12)-2-Glu(12) (b) Glu(12)-2-Glu(12)+10 mM KI.

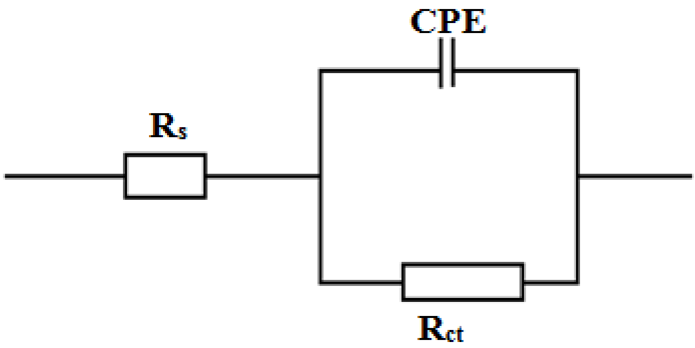

Figure S4. Equivalent circuit model used to fit the impedance measurement data for MS in 1M HCl (R_s = solution resistance, R_{ct} = charge-transfer resistance, and *CPE* = constant phase element).

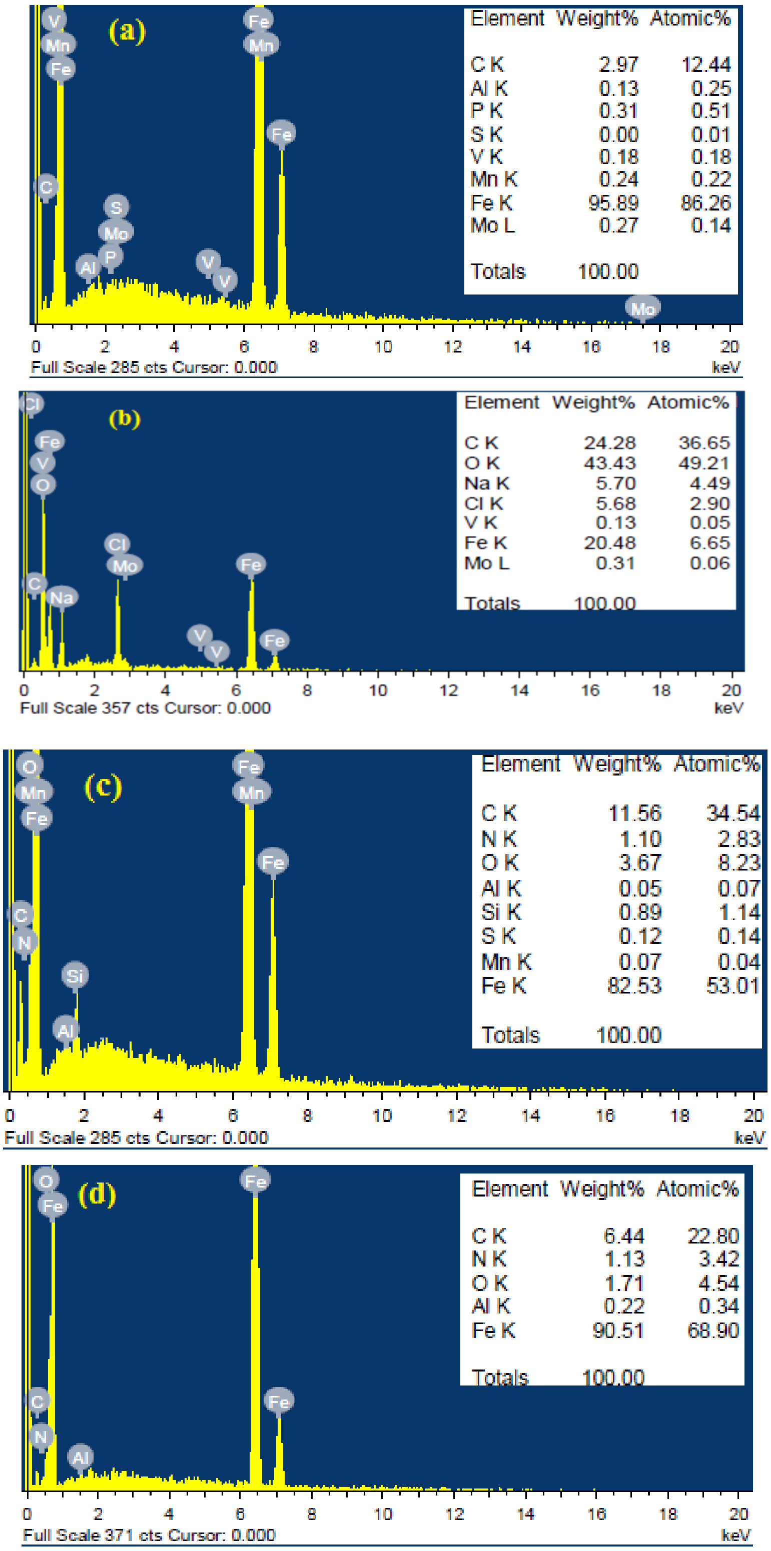

Figure S5. EDAX images of the MS after 6 h immersion: (a) polished MS prior to immersion, (b) uninhibited solution, (c) in 3.5% NaCl solution with 2.5×10^{-3} mM Glu(12)-2-Glu(12) (d) in 3.5% NaCl solution with 2.5×10^{-3} mM Glu(12)-2-Glu(12)+10 mM KI.

Table S1: Slopes and regression coefficients (R^2) (a) Langmuir (b) Temkin (c) Frumkin and (d) Freundlich, adsorption isotherms plots for investigated inhibitor.

Langmuir adsorption isotherm		Temkin adsorption isotherm		Frumkin adsorption isotherm		Freundlich adsorption isotherm	
slope	R^2	slope	R^2	slope	R^2	slope	R^2
1.427	0.998	0.180	0.987	8.367	0.973	0.169	0.969
1.251	0.997	0.198	0.976	6.957	0.936	0.161	0.977
1.209	0.997	0.190	0.986	7.438	0.958	0.142	0.990
1.076	0.996	0.209	0.978	5.392	0.861	0.141	0.985
